# Laminar Flame Speeds of Premixed Dimethoxymethane/Air Flames at Elevated Temperatures and Pressures

**DOI:** 10.1021/acsomega.6c06957

**Published:** 2026-10-12

**Authors:** Sebastian Galeano Herrera, Ahmed Barain, Berk Can Duva, Elisa Toulson

**Affiliations:** † Alternative Fuels and Combustion Laboratory, Department of Mechanical Engineering, 468728 Michigan State University, East Lansing, Michigan 48824-1312, United States; ‡ Rolls-Royce Solutions America Inc., Graniteville, South Carolina 29829, United States

## Abstract

The world’s increasing energy demand combined with the continuous depletion of fossil fuel reserves and growing interest in reducing greenhouse gas emissions has spurred research on new alternative fuels that can partially satisfy the energy demand while mitigating air pollution. In this regard, dimethoxymethane (DMM), an oxygenated fuel that can be produced from renewable sources, is a promising alternative fuel that can be used in diesel blends in combustion engines. This research presents DMM laminar flame speed measurements of outwardly propagating flames, which were carried out in an optically accessible constant-volume chamber at several temperatures (373, 423, and 473 K) and pressures (1, 3, and 5 bar), for equivalence ratios from 0.7 to 1.7. The experimental results were compared against one-dimensional laminar premixed flame simulations from Converge CFD using detailed kinetic mechanisms. At 1 bar, the maximum DMM laminar flame speeds were found to be 63.4, 77.5, and 91.8 cm/s at 373, 423, and 473 K, respectively, at an equivalence ratio of 1.2. At 373 K and 3 and 5 bar, the maximun flame speeds were 49.8 and 43.2 cm/s at equivalence ratios of 1.2 and 1.1, respectively.

## Graphical abstract

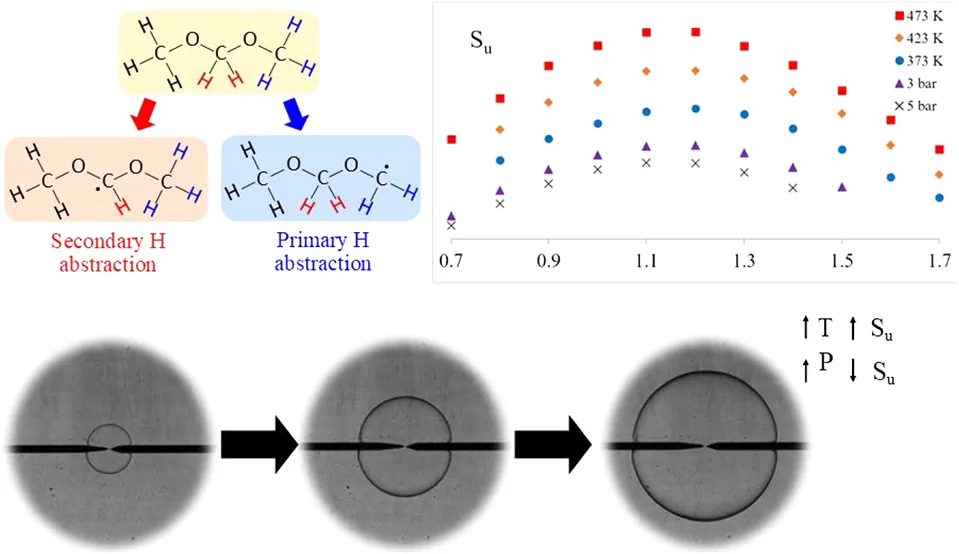

## 1 Introduction

Combustion has long been the main source of energy worldwide, with combustion of coal, oil, and natural gas accounting for more than 86% of the world’s energy supply.^1^ In addition, the global energy demand is expected to grow by 34% from 673 exajoules in 2022 to 902 exajoules by 2050, driven by industrialization, demographic growth, and rapid economic development.^2^ Moreover, despite the record level of clean energy available globally, two-thirds of the increase in energy demand in 2023 was still met by fossil fuels.^3^ As a result, fossil fuel combustion is expected to be a major energy source for the foreseeable future.

The widespread use of combustion also has an environmental impact, with energy-related CO_2_ emissions reaching 37.7 gigatons (Gt) in 2023.^3^ Additionally, 2023 and 2024 were among the hottest years in recorded history, with extreme heat events occurring worldwide and consequent harmful effects on infrastructure, services, vulnerable population, and the natural environment.^4^

The increasing concern for environmental protection, together with depleting petroleum deposits, is driving research on alternative fuels.^5^ As a result, several alternative fuels, such as hydrogen, ammonia, ethanol, biodiesel, vegetable bio-oils, and oxymethylene ethers, have been considered feasible substitutes for hydrocarbon fuels.^5–10^

Oxymethylene ethers (OME) are ethers with the general chemical formula CH_3_–O–(CH_2_–O)_*n*_–CH_3_ and are promising alternative fuels^9^ that can be synthesized from renewable energy sources^11,12^ and help reduce soot emissions due to their lack of C–C bonds and high oxygen content.^13,14^ Dimethoxymethane (DMM), CH_3_–O–CH_2_–O–CH_3_, also known as methylal, is the smallest oxymethylene ether and is usually used in diesel blends or with additives.^13,14^

Several studies have been carried out on DMM speciation^15–22^ and ignition delay.^14,23,24^ However, laminar flame speed measurements remain scarce. Laminar flame speed is a fundamental combustion parameter used to develop and validate detailed chemical kinetic mechanisms.^25,26^

Some studies have been conducted on the laminar flame speed of DMM, as shown in Table 1. Gillespie^27^ measured DMM laminar flame speed in a heat flux burner at 1 atm and several temperatures (298, 328, and 358 K). The equivalence ratio analyzed ranged from 0.5 to 2. The results showed that the maximum laminar flame speed values are 45.6, 52.7, and 60.4 cm/s at 298, 328, and 358 K, respectively, at an equivalence ratio of 1.2.

**Table 1:** Summary of Previous Studies on DMM

Experimental method	Conditions	Fuel	Reference
Heat flux burner	*P* = 1 atm, *T* = 298 K	DMM	Gillespie et al.^27^
Heat flux burner	*P* = 1 atm, *T* = 328 K	DMM	Gillespie et al.^27^
Heat flux burner	*P* = 1 atm, *T* = 358 K	DMM	Gillespie et al.^27^
Heat flux burner and constant-volume vessel	*P* = 1 bar, *T* = 298 K	DMM	Shrestha et al.^13^
Heat flux burner and constant-volume vessel	*P* = 3 bar, *T* = 298 K	DMM	Shrestha et al.^13^
Heat flux burner and constant-volume vessel	*P* = 5 bar, *T* = 298 K	DMM	Shrestha et al.^13^
Heat flux burner and constant-volume vessel	*P* = 1 bar, *T* = 373 K	DMM	Shrestha et al.^13^
Heat flux burner	*P* = 1 atm, *T* = 323 K, *dilution* = 0–70%	DMM (diluted with N_2_)	Eckart et al.^28^
Heat flux burner	*P* = 1 atm, *T* = 373 K	DMM	Eckart et al.^28^
Constant-volume vessel	*P* = 1 bar, *T* = 298 K, *X*_*DMM*_ = 0.2–0.6	DMM/NH_3_	Elbaz et al.^29^
Constant-volume vessel	*P* = 1 bar, *T* = 298 K, *X*_*DMM*_ = 0–1	DMM/NH_3_	Sun et al.^30^
Constant-volume vessel	*P* = 1 bar, *T* = 373 K, *X*_*DMM*_ = 0.2	DMM/NH_3_	Sun et al.^30^
Constant-volume vessel	*P* = 1 bar, *T* = 473 K, *X*_*DMM*_ = 0.2	DMM/NH_3_	Sun et al.^30^
Constant-volume vessel	*P* = 2 bar, *T* = 373 K, *X*_*DMM*_ = 0.2	DMM/NH_3_	Sun et al.^30^
Constant-volume vessel	*P* = 4 bar, *T* = 373 K, *X*_*DMM*_ = 0.2	DMM/NH_3_	Sun et al.^30^

Shrestha et al.^13^ measured the laminar flame speed of DMM using both a heat-flux burner and a constant-volume spherical vessel at 1 bar and temperatures ranging from 298 to 373 K. In addition, at 298 K, several measurements were done at different pressures (1, 3, and 5 bar). The equivalence ratio ranged from 0.6 to 1.8. The experimental measurements were compared to numerical simulations using a new chemical kinetic mechanism for DMM oxidation developed by the authors that includes *NO*_*x*_ chemistry. The results indicated that at 1 bar, the peak flame speed values were 46.8 and 63.5 cm/s at 298 and 373 K, respectively, at an equivalence ratio of 1.2.

Furthermore, in Shrestha et al.,^13^ at 298 K, when the pressure was increased to 3 and 5 bar, the maximum flame speeds were 36.8 and 31.8 cm/s, respectively, showing a clear reduction in flame speed with increasing pressure. The influence of pressure on the reduction of the laminar flame speed decreased at higher pressures. Overall, the predictions from the model developed by Shrestha et al.^13^ showed good agreement with experimental values. At 1 bar and temperatures of 298 and 373 K, the simulations closely matched the experimental results. Similarly, at higher pressures (3 and 5 bar), the simulated results were close to the measured values under lean and stoichiometric conditions. Nonetheless, for rich conditions, some deviations were observed with the model underpredicting the experimental results. The experimental results at 298 K and 1 bar published by Gillespie^27^ and Shrestha et al.^13^ show good agreement with each other.

Eckart et al.^28^ also studied the DMM laminar flame speed in a heat-flux burner setup. At 1 atm, the temperature varied from 298 to 373 K and the equivalence ratio varied from 0.7 to 1.6. The measured data were compared with numerical simulations using the kinetic mechanism developed by Jacobs et al.^14^ and Ren et al.^31^ At 323 K, the experimental results indicated that the laminar flame speed of pure DMM reached a maximum of approximately 50 cm/s at an equivalence ratio of 1.2. The Jacobs et al.^14^ mechanism slightly overpredicted the experimental results but followed a similar trend, with excellent agreement at equivalence ratios of 1.3 to 1.6. The Ren et al.^31^ mechanism, on the other hand, significantly overestimated the measured values for equivalence ratios above 0.9. This may be a result of the Ren et al.^31^ mechanism being validated against results from oxymethylene ether-3 (OME_3_) combustion and containing only a small fraction of the chemical species included in the Jacobs et al.^14^ mechanism.

Elbaz et al.^29^ measured the laminar flame speed of DMM in a constant-volume vessel at 1 bar and 298 K. It was demonstrated that DMM laminar flame speed varies from 30 cm/s at an equivalence ratio of 0.8 to a maximum value of 47 cm/s at an equivalence ratio of 1.2, closely matching the results published by Gillespie^27^ under the same conditions.

Sun et al.^30^ measured neat DMM laminar flame speeds at 298 K and 1 bar in a constant-volume vessel and compared the results with data published by other authors (Elbaz et al.,^29^ Gillespie,^27^ and Shrestha et al.)^13^ Generally, the results showed good agreement with the data published in the literature under lean and stoichiometric conditions, with a peak laminar flame speed of 45.5 cm/s at an equivalence ratio of 1.1. However, in the rich region, some differences were observed. The values measured by Sun et al.^30^ are lower than those published by Elbaz et al.^29^ and Gillespie^27^ and higher than those published by Shrestha et al.^13^

As shown in the preceding literature review, previous studies measured the laminar flame speed at relatively lower temperatures. However, engine-relevant conditions are usually associated with higher temperatures and pressures.^32^ Therefore, the aim of this work is to measure DMM laminar flame speed in a constant-volume combustion chamber (CVCC) under new experimental conditions that have not been studied before, particularly elevated temperatures and pressures. In this regard, DMM laminar flame speeds were measured at 1 bar and temperatures of 373, 423, and 473 K. In addition, at 373 and 423 K, pressures of 3 and 5 bar were used. The equivalence ratios considered varied from 0.7 to 1.7.

## 2 Experimental Setup and Procedure

### 2.1 The Constant-Volume Combustion Chamber

The laminar flame speed experiments were carried out in the constant-volume combustion chamber (CVCC), as shown in Figure 1. The CVCC consists of a main cylindrical body fabricated from 304 stainless steel. Its outer diameter (OD), inner diameter (ID), length, and inner volume are 48.26 cm, 30.48 cm, 45.72 cm, and 22.4 L, respectively. Two fused-quartz windows are connected to each end of the main body and allow for observation of the flame inside the chamber. The windows are secured with end window flanges (OD = 30.48 cm, ID= 15.24 cm, and thickness 5.08 cm).

**Figure 1:**
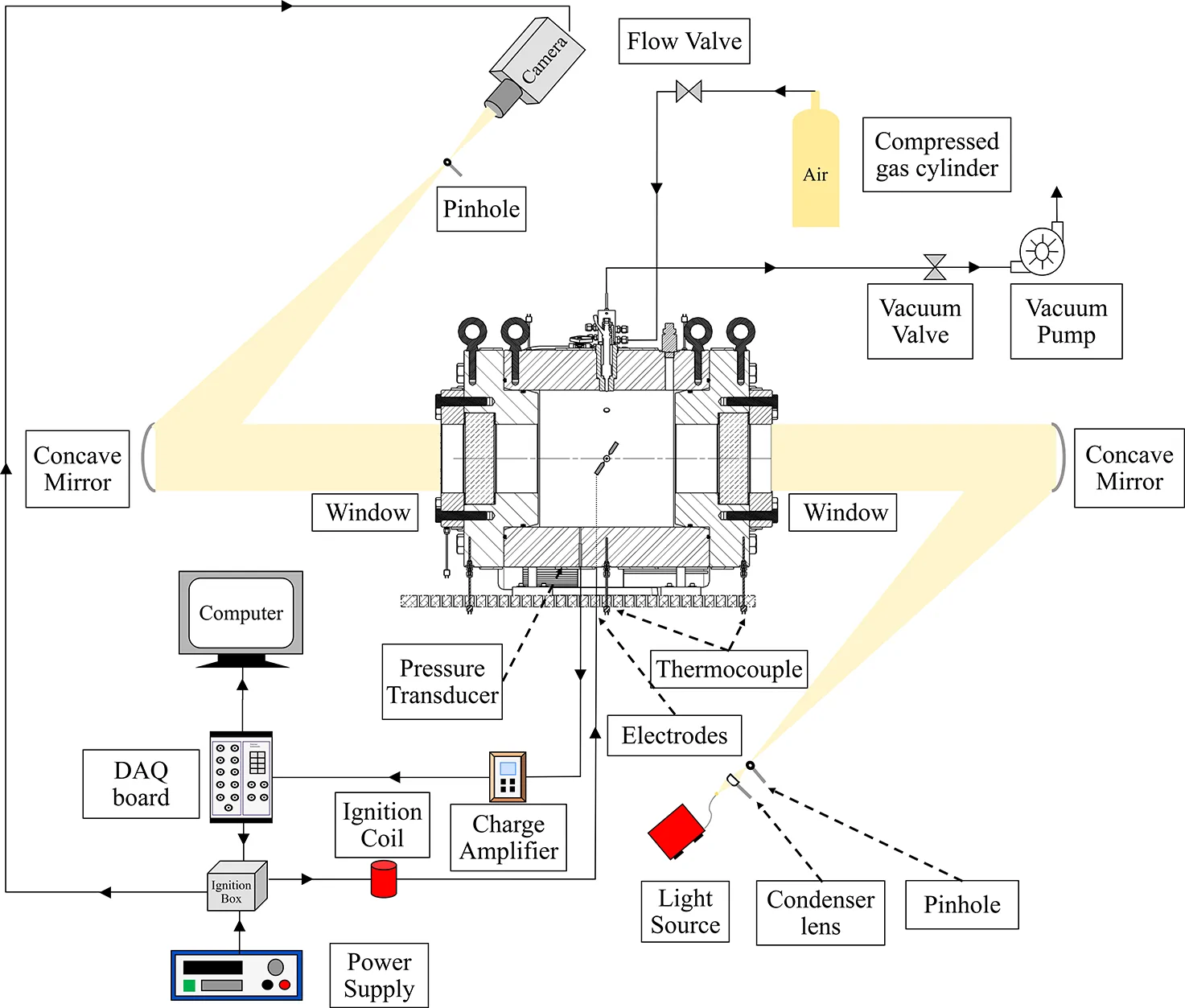
Constant-volume combustion chamber and Schlieren imaging system.

The combustion chamber was heated using six heating bands with heating power varying from 0.35 kW to 1.75 kW and controlled by a heating unit with integrated cascade and high-limit controllers. Six type-T thermocouples manufactured by Omega were installed in the vessel to collect temperature data. These thermocouples were inserted to a depth of three-quarters of the vessel wall thickness and were used to measure the wall temperature. An additional thermocouple was located all the way through the inner wall thickness and was used to measure the unburned gas temperature inside the combustion chamber. A fiberglass insulating jacket was used to reduce heat losses to the surroundings and to keep temperature fluctuations less than ±3 ^◦^C.

An Edwards RV5 vacuum pump was utilized before and after each test to empty the vessel contents, removing the products of combustion and the unburned reactants. The combustion chamber was then flushed with air to ensure the removal of volatile substances and soot before each test. Compressed gas cylinders were used to store dry, high-purity air and nitrogen supplied by Airgas.

The mixture preparation process consisted of filling the combustion chamber with each reactant (fuel and air) from the lowest to highest concentration. The partial pressure of each mixture component before combustion was measured with an Omega MM-series absolute pressure transducer. A Kistler 6125C piezoelectric pressure sensor was used to measure the pressure traces after the start of the combustion.

The fuel was supplied by Fisher Scientific in 2.5 liter amber glass bottles with a 99.5% purity and water content lower than 0.03%. Before each test, the vessel was vacuumed to a pressure lower than 0.015 bar, and then the fuel was injected. The liquid fuel was stored in a fuel accumulator that consists of a cylindrical metal container with a moving piston head that separates two inner chambers: the lower chamber that contains high-pressure nitrogen and the upper chamber that contains the liquid fuel. Nitrogen at 500 psi was supplied to the lower chamber to compress the liquid fuel and inject it into the constant-volume chamber through a Bosch injector. The fuel accumulator was connected to the Bosch injector with a stainless-steel high-pressure hose. The fuel line and fuel accumulator were kept at room temperature. The fuel injector was calibrated by mass and partial pressure to ensure proper fuel delivery and evaporation.

Dimethoxymethane is a volatile liquid with a boiling point of 42 °C at 1 atm.^33^ Thus, due to the low initial pressure in the combustion chamber (<0.015 bar) and high temperature (≥100 °C), evaporation of the liquid fuel was easily achieved. During the entire charge preparation process, no condensation, mist, or droplets were observed on the windows or the wall surfaces. The evaporation time was 2 min. After adding fuel, air was fed to the combustion chamber through a gas manifold, which is separated from the combustion chamber by a needle valve with a regulating stem (maximum pressure of 512 bar at 505 K). This valve is kept closed before ignition to avoid high pressures in the manifold.

An electric fan located on top of the CVCC was turned on for 5 min to stir the mixture and ensure proper mixing and uniform temperature at a speed of 400 rpm. In addition, the fan was turned off 2 min before combustion to avoid vibrations and disturbances of the flame.^34,35^ Further increasing the evaporation and mixing times was not found to affect the laminar flame speed values.

The reactant gaseous mixtures were prepared using the partial-pressure method, assuming all components are ideal gases. Based on the chemical reaction for 1 mol of DMM (eq 1), the defined initial pressure *P*_*initial*_, and the equivalence ratio *ϕ*, the partial pressures of fuel and air can be calculated using the following equations:

1 $$C_{3}H_{8}O_{2}+4\left(O_{2}+3.76N_{2}\right)\to 3CO_{2}+4H_{2}O+15.04N_{2}$$

2 $$P_{fuel}=\frac{P_{initial}}{1+\left(\frac{MW_{fuel}}{MW_{air}}\right)\times \frac{1}{\phi }\times {\left(\frac{A}{F}\right)}_{\phi =1}}$$

3 $$P_{air}=P_{fuel}\times \frac{MW_{fuel}}{MW_{air}}\times \frac{1}{\phi }\times {\left(\frac{A}{F}\right)}_{\phi =1}$$

The air/fuel mixture was ignited by a spark generated using two tungsten electrodes (OD = 3 mm, gap = 2 mm) located at the center of the combustion chamber. One electrode was used as the ground, and the other was connected to the ignition coil. The ignition system consisted of a 13.8 V direct current (DC) power supply, an ignition circuit, and an ignition coil. A LabVIEW program was used to generate the trigger signal to activate the ignition system.

A high-speed camera (Photron SA5) was used at different frame speeds (20,000 to 42,000 frames per second) to capture Schlieren images of the expanding flame front. As shown in Figure 1, a z-type Schlieren setup was used to capture the flame. The light beam was generated by a Thorlabs OSL 2 tungsten-halogen light source and was aimed at an aspheric condenser lens to reduce noise. The light beam then goes through an iris diaphragm to reduce excess light. A 15.24 cm parabolic concave mirror reflects and collimates the light beam, which goes through the combustion chamber and is refracted by the difference in the gas density inside the chamber. Another concave mirror reflects the light coming from the combustion chamber and focuses it on an iris diaphragm in front of the high-speed camera.

The experimental conditions (temperature, pressure, and equivalence ratio) considered in this work are summarized in Table 2.

**Table 2:** Experimental Temperature, Pressure, and Equivalence Ratio for the Present Study

Pressure (bar)	Temperature (K)	Equivalence ratio
1	373	0.7–1.7
3		
5		
1	423	
3		
5		
1	473	

### 2.2 Premixed Laminar Flame Speed Calculation

As can be observed in Figure 2, the minimum and maximum flame radii considered for the laminar flame speed measurement were carefully selected to avoid the spark effect, cellularity, and a significant pressure rise inside the vessel (<5% of *P*_*initial*_).^35^ Moreover, the radiation- and confinement-induced inward flow effects are reduced when the flame occupies less than 25% of the total chamber volume,^35^ which is equivalent to a 4.5 cm flame radius.

**Figure 2:**
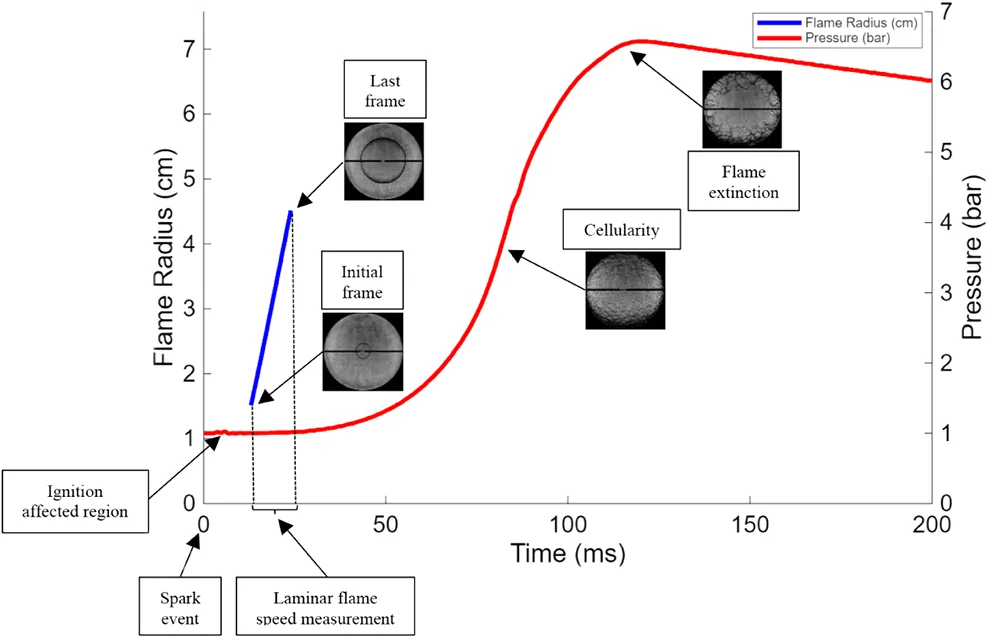
Pressure traces and flame radius vs time, and Schlieren images of the flame front for *ϕ* = 0.9 at 1 bar and 373 K.

Image processing was performed on the Schlieren images acquired after each test. An in-house MATLAB image-processing code was used to detect the flame edge and to calculate the radius of the flame front based on the Canny edge detection technique.

The electrodes and the background were removed from each image to extract the flame front boundary, and the distance from the electrode centerline to the flame edge was used to compute the flame volume from which an equivalent spherical radius was calculated.

The stretched laminar flame speed *S*_*b*_ relative to the burned gas was calculated from the time rate of change of the flame radius *R*_*f*_ as indicated in eq 4.

4 $$S_{b}=\frac{dR_{f}}{dt}$$

The speed of a spherically propagating flame is affected by the flame curvature and strain, represented by flame stretch rate (*κ*),^36,37^ which is defined as the rate of change of flame surface area per unit area (eq 5) and can also be expressed as eq 6 for spherical flames.

5 $$\kappa =\frac{1}{A}\times \frac{dA}{dt}$$

6 $$\kappa =2\times \frac{S_{b}}{R_{f}}$$

Since both the stretched laminar flame speed (*S*_*b*_) and the flame radius (*R*_*f*_) are positive, the flame stretch is always positive for expanding flames. Moreover, as the flame expands, the stretch effect decreases. Two correlations were used to extrapolate the stretched flame speed to a zero-stretch value (or infinite radius, since κ → 0 when *R*_*f*_ → ∞) and calculate the unstretched burned gas laminar flame speed $S_{b}^{◦}$ , the nonlinear correlation proposed by Kelley and Law^38^ expressed for a spherically expanding flame (eq 7), and the correlation originally presented by Markstein^39^ and revised by Chen^36^ (eq 8), where *L*_*b*_ is the Markstein length.

7 $$\frac{S_{b}}{S_{b}^{◦}}\times \ln\left(\frac{S_{b}}{S_{b}^{◦}}\right)=-\frac{2\times L_{b}}{R_{f}}$$

8 $$S_{b}=S_{b}^{◦}-S_{b}^{◦}\times L_{b}\times \frac{2}{R_{f}}$$

It was demonstrated that the equation proposed by Markstein^39^ (eq 8) is more accurate for mixtures with *L*_*b*_ > 0 while the equation presented by Kelley and Law^38^ (eq 7) is more precise when *L*_*b*_ < 0.^36,40–42^ Hence, depending on the mixture’s Markstein length, eq 7 or 8 is used for the extrapolation.

Finally, the unstretched laminar burning velocity relative to the unburned gas $S_{u}^{◦}$ can be calculated by applying the continuity equation to the flame surface (eq 9) where *ρ*_*u*_ and *ρ*_*b*_ are the unburned gas density and burned gas density, respectively.

9 $$S_{u}^{◦}=S_{b}^{◦}\times \frac{\rho_{b}}{\rho_{u}}$$

The constant volume combustion chamber and the laminar flame speed calculation methodology were validated by Duva and Toulson^40^ for H_2_/air flames at 1 bar, 298 K, and *ϕ* = 0.7–4. The experimental results were compared with data reported by Beeckmann and Pitsch,^43^ Krejci et al.,^44^ Dong et al.,^45^ and Kuznetsov et al.,^46^ showing in general good agreement.

### 2.3 Laminar Flame Speed Simulation and Chemical Kinetic Mechanism

The chemistry module in CONVERGE CFD^47,48^ was used to perform 1D laminar flame speed simulations and compare numerical results with experimental measurements. In Section 3.1, the experimental results are compared to simulations using the Jacobs et al.^14^ mechanism, consisting of 530 species and 2,889 chemical reactions. In addition, in Section 3.2, additional comparisons are carried out against other chemical kinetic models available in the literature and summarized in Table 3.

**Table 3:** Chemical Kinetic Mechanisms Used for 1D Laminar Flame Speed Simulations

Mechanism	Fuel	Species and reactions
Jacobs et al.^14^	DMM	530 species and 2,889 reactions
Shrestha et al.^13^	DMM/DME	172 species and 1,375 reactions
García-Oliver et al.^12^	n-dodecane/DMM	121 species and 678 reactions
Ren et al.^31^	PRF-PODE_3_	145 species and 585 reactions
Sun et al.^22^	Ethane/DMM/DMC	283 species and 1,715 reactions

Jacobs et al.^14^ developed a kinetic mechanism for DMM oxidation, whereas Shrestha et al.^13^ created a kinetic model for the combustion of DMM and dimethyl ether (DME).

García-Oliver et al.^12^ modeled the combustion of hydrotreated vegetable oil (HVO) and DMM, considering n-dodecane as an HVO surrogate. For that reason, they combined the mechanism from Yao et al.^49^ for n-dodecane with a reduced mechanism for DMM from Jacobs et al.^14^ to obtain a final kinetic model with 121 species and 678 chemical reactions.

Ren et al.^31^ developed a kinetic mechanism for the combustion of a primary reference fuel (PRF) with polyoxymethylene dimethyl ether (PODE) in internal combustion engines. Even though this mechanism was optimized for PODE_3_ CH_3_O­(CH_2_O)_3_CH_3_, it also contains DMM in the species list.

Sun et al.^22^ proposed a kinetic mechanism for premixed flames containing ethane, DMM, and dimethyl carbonate (DMC). The results were validated against concentration profiles for flame species measured by molecular-beam mass spectroscopy.

In order to quantify the deviation of each kinetic model with respect to the experimental laminar flame speed values, the error is calculated as indicated in eq 10:

10 $$Error_{sim}=\frac{\left|S_{u}^{◦}-S_{u_{sim}}^{◦}\right|}{S_{u}^{◦}}$$

where $S_{u}^{◦}$ and $S_{u_{sim}}^{◦}$ are the experimental and simulated laminar flame speed values, respectively.

The parameters used in the laminar flame speed simulations in CONVERGE are shown in Table 4. Grid stability and convergence were tested by further increasing the number of grid points and reducing tolerance limits. The results indicate that laminar flame speed values change by only less than 1% after these adjustments.

**Table 4:** Parameters for 1D Laminar Flame Speed Simulations in CONVERGE CFD

Parameter	Value/Method
Initial number of grid points	50
Final number of grid points after refinement	600–700
Normalized slope criteria for refinement	0.03
Normalized curvature criteria for coarsening	0.03
ODE solver	Iterative SuperLU
Transport properties model	Mixture-averaged

For the reaction pathway analysis, a 1D laminar flame speed simulation was performed using the Jacobs et al.^14^ mechanism in CHEMKIN-Pro 2024 R2,^50,51^ and the Reaction Path Analyzer (RPA) was used to generate a visual representation of the key species involved in the DMM breakdown and the chemical reactions that deplete or produce them.

It is worth noting that, in the present work, the laminar flame speed experimental results are compared against numerical values from CONVERGE CFD simulations. Nevertheless, both CONVERGE CFD and CHEMKIN-Pro 2024 R2 achieve similar flame speed results (around a 3.6% difference at 373 K, 1 bar, and *ϕ* = 1). In addition, for both simulations in CONVERGE CFD and CHEMKIN-Pro, the same kinetic mechanism, thermodynamic, and transport properties files from Jacobs et al.^14^ were used.

### 2.4 Flame Stability Analysis

The smooth, stable propagation of a flame can become unstable, with wrinkles, cracks, and cells extending on the flame surface as it expands.^52,53^ These cellular instabilities are generated by three intrinsic mechanisms: (1) diffusional-thermal, (2) hydrodynamic (also known as the Darrieus–Landau instability), and (3) buoyancy-driven instability.^52,54^

The diffusional-thermal instabilities are caused by a difference in the diffusion rates of heat and species (mass).^53,55^ This imbalance induces a perturbation that makes the cells grow, leading to an unstable flame. This effect can be characterized by the Lewis number (eq 11), which is the ratio of thermal to mass diffusivities.^56^ When *Le* is lower than 1, mass transport of reactant by diffusion into the reaction zone occurs faster than thermal conduction from the flame into the unburned gas.^54,57^ As a result, temperature and flame speed increase, making the wrinkles more unstable. Conversely, if *Le* is higher than 1, heat diffusion is dominant and the flame is thermodiffusively stable.^52,53,55,57–59^

11 $$Le=\frac{\alpha }{D_{ij}}$$

The effective Lewis number (eq 12) is defined as a weighted average of the excess reactant Lewis number (*Le*_*E*_) and deficient reactant Lewis number (*Le*_*D*_) where the variable ξ is the mixture strength (eq 13) and *Ze* is the Zel’dovich number.^53,54^

12 $$Le_{eff}=1+\frac{\left(Le_{E}-1\right)+\left(Le_{D}-1\right)\times \xi }{1+\xi }$$

13 ξ=1+Ze×(φ−1)

The parameter *φ* is defined as *φ* = 1/*ϕ* for *ϕ* < 1 (lean mixtures) while *φ* = *ϕ* for *ϕ* > 1 (rich mixtures). The Zel’dovich number is calculated based on the overall activation energy *E*_*a*_, universal gas constant *R*, adiabatic flame temperature *T_ad_,* and unburned gas temperature *T*_*u*_.

14 $$Ze=E_{a}\times \frac{\left(T_{ad}-T_{u}\right)}{RT_{ad}^{2}}$$

The overall activation energy *E*_*a*_ is empirically calculated using the equation indicated by Egolfopoulos and Law^60^ (eq 15). The values for adiabatic flame temperature *T*_*ad*_ and unstretched unburned gas flame speed $S_{u}^{◦}$ are numerically determined via 1D simulations in CONVERGE at different initial temperatures.

15 $$E_{a}=-2\times R\times \frac{\partial ln\left(\rho_{u}\times S_{u}^{◦}\right)}{\partial \left(T_{ad}^{-1}\right)}$$

Hydrodynamic instabilities occur due to thermal expansion of the gas throughout the flame due to the density jump at the flame front. As a result, the flow accelerates across the flame and wrinkles become unstable.^52,57^ This instability is characterized by the density variation across the reaction zone, namely, the density ratio (eq 16), and the flame thickness δ_*tg*_ (defined based on the thermal gradient in eq 17). Flames that are thin with a significant density jump are more unstable and susceptible to hydrodynamic instabilities, even at small radii.^54^

16 $$\sigma =\frac{\rho_{u}}{\rho_{b}}$$

17 $$\delta_{tg}=\frac{T_{ad}-T_{u}}{max\left(dT/dx\right)}$$

Another parameter that characterizes flame stability is the Markstein length *L*_*b*_ (calculated from eqs 7 and 8) since it describes the flame response to stretch. At large stretch rates (small flame radius), the strong flame stretch rate plays a stabilizing role for the flame, and flame instabilities are suppressed.^59,61^ Nonetheless, as the flame propagates, the stretch rate is reduced, and its stabilization effect weakens; hence, the flame is destabilized by thermodiffusive and hydrodynamic instabilities.^59^ Moreover, the Markstein length is positive when the flame speed decreases with the stretch rate. Conversely, it is negative when the flame speed increases with the stretch rate. Thus, the flame response to stretch plays a destabilizing role when *L*_b_ < 0.^59^

Finally, the buoyancy-driven instability, also known as the Rayleigh–Taylor instability, arises from body forces when the density decreases in the direction of gravity. This occurs during the propagation of an upwardly expanding flame, where the higher-density unburned mixture lies above the lighter burned gas, making the flame buoyantly unstable.^52^

In the present work, the stability analysis is focused on the first two mechanisms, diffusional-thermal and hydrodynamic, since the DMM flames under the studied conditions do not exhibit buoyancy effects.

### 2.5 Uncertainty Analysis

The total uncertainty in the laminar flame speed measurement is estimated by^53,62^

18 $$\delta_{S_{L}}=\sqrt{B_{T}^{2}+B_{\phi }^{2}+{\left(\frac{t_{0.95}\times S_{S_{L}}}{\sqrt{N}}\right)}^{2}}$$

where *B*_*T*_ and *B*_*ϕ*_ are the systematic uncertainties due to variations in temperature (±3 °C) and equivalence ratio (generated by variations in pressure of ±0.001 bar). These terms are estimated by simulations in CONVERGE with the modified temperatures and equivalence ratios. The term $\left(t_{0.95}\times S_{S_{L}}\right)/\sqrt{N}$ is the random uncertainty where the Student’s *t* value for a 95% confidence level is *t*_0.95_ = 4.3 and $S_{S_{L}}$ is the standard deviation of the experiments under the same condition. Each condition is repeated 3 times (*N* = 3).

## 3 Results and Discussion

### 3.1 Flame Morphology and Laminar Flame Speed

The flame morphology for mixtures at *ϕ* = 1 and 1 bar, at temperatures of 373, 423, and 473 K, is shown in Figure 3. The flames are smooth, with no cellularity, wrinkles, or buoyancy affecting their propagation. It can be observed that increasing the initial temperature accelerates the flame propagation and the flame reaches the maximum radius sooner.

**Figure 3:**
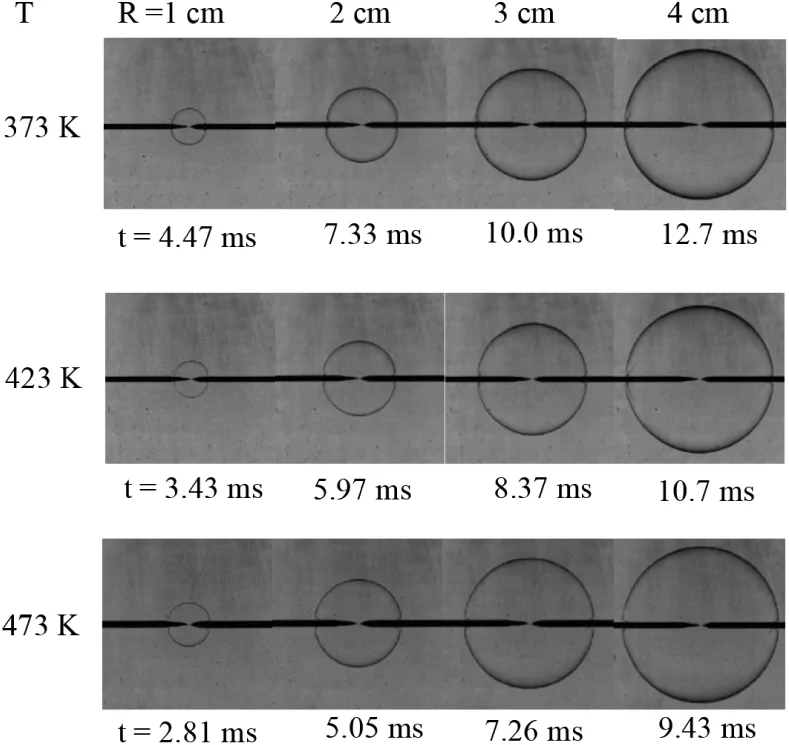
Flame morphology versus time for *ϕ* = 1 at 1 bar and temperatures 373, 423, and 473 K.

The flame morphology at 3 and 5 bar is shown in Figure 4 (373 K) and Figure 5 (423 K). At higher pressures, the flame is slower and takes a longer time to reach the final radius. Moreover, because of the increased density change, the flame looks darker in the Schlieren images. At higher pressures (3 and 5 bar), the flames are more unstable and small wrinkles and cracks start to develop on the flame surface as the flame expands. In particular, at 5 bar, more cells and cracks can be observed on the entire flame surface when the radius reaches 4 cm. These wrinkles affect the flame surface, increasing the flame area and, thus, the flame speed. For this reason, the radius window was adjusted under each condition to avoid the effect of cellularity on the flame speed.

**Figure 4:**
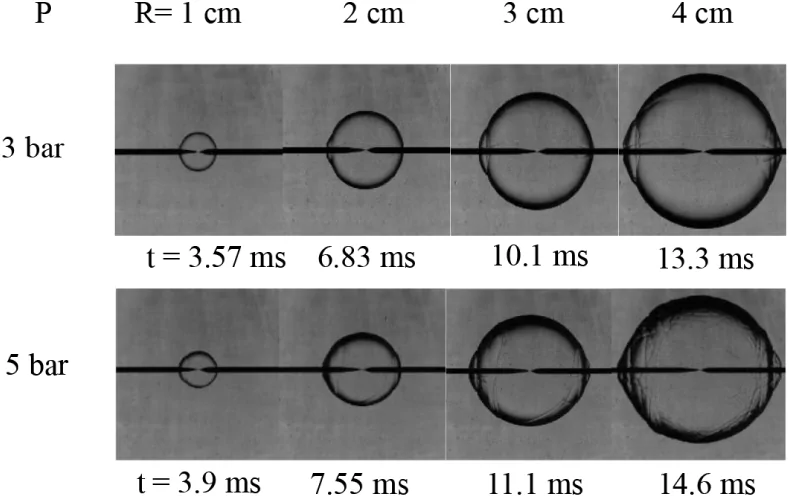
Flame morphology versus time at 373 K, *ϕ* = 1, and pressures of 3 and 5 bar.

**Figure 5:**
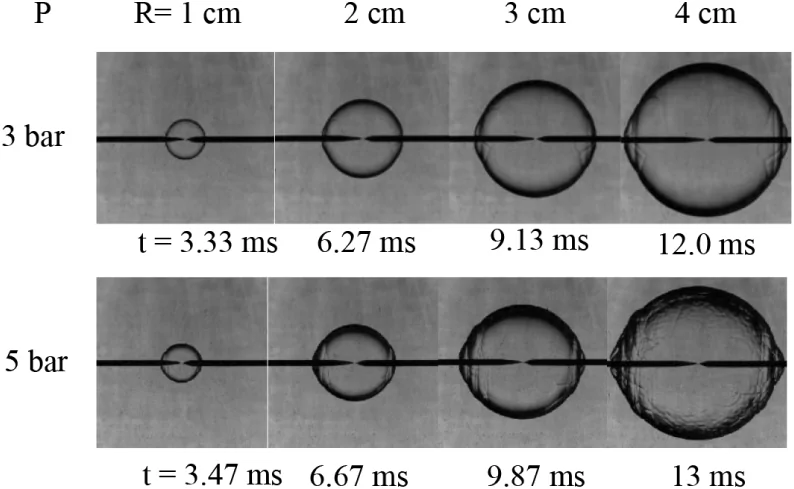
Flame morphology versus time at 423 K, *ϕ* = 1, and pressures of 3 and 5 bar.

As shown in the previous figures, the flame front is clearly observed because of the high contrast between the dark flame and the background. In addition, this enhances the detection of the flame front by MATLAB code.

The radius windows used in the image processing were adjusted according to the conditions and the presence of cellularity, particularly at high pressures. At 1 bar, and 373, 423, and 473 K, the flame speed calculation was not affected by cellularity, and thus, a large radius window was used (1–4.5 cm). For a stoichiometric mixture, the stretched laminar flame speed is shown in Figure 6 at 1 bar. The increase in temperature increases the stretched laminar flame speed from 379 cm/s at 373 K to 465 cm/s at 473 K. At the beginning of flame propagation (small radii), the energy from the spark used to ignite the mixture causes a minor oscillation in the flame speed, which differs from the stable values and is not considered in the extrapolation to calculate the unstretched flame speed.

**Figure 6:**
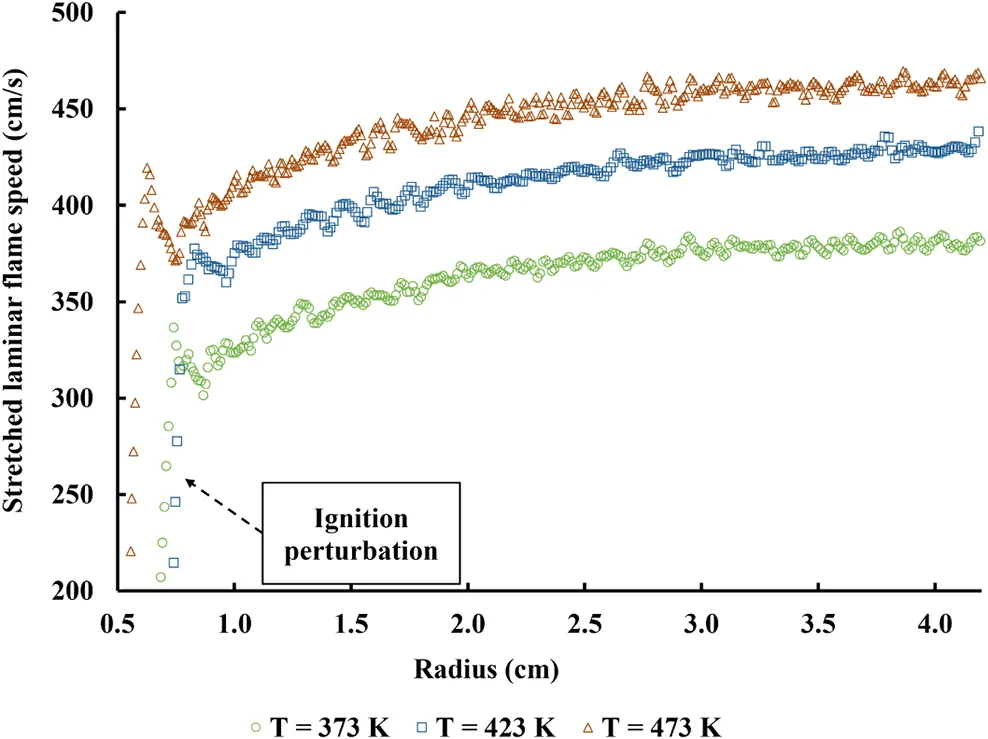
Stretched burned gas flame speed versus flame radius for *ϕ* = 1, *T* = 373, 423, and 473 K, *P* = 1 bar.

The stretched laminar flame speed was extrapolated to a zero-stretch value (or 2/*Radius* = 0) as shown in Figure 7 for flames at 1 bar and *ϕ* = 1. For all temperatures, a negative slope in the *S*_*b*_ vs 2/*Radius* plot is observed. For small, highly stretched flames (high values of 2/*Radius*), the flame speed is slower, while larger, slightly stretched flames (small values of 2/*Radius*) show faster flame propagation. This linear correlation is not maintained at small radii, where the spark energy disturbance occurred. Thus, the ignition-affected region was not included in the extrapolation.

**Figure 7:**
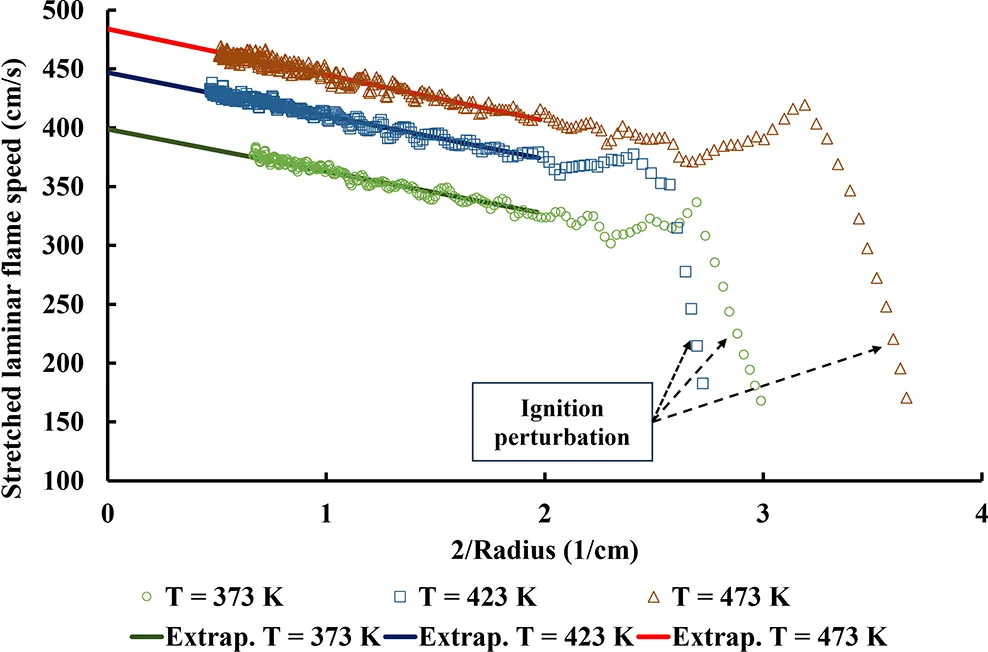
Stretched burned gas flame speed versus 2/*Radius* for *ϕ* = 1, *T* = 373, 423, and 473 K, *P* = 1 bar.

At 3 bar and 373 K, for equivalence ratios from 0.7 to 1.1, a radius range from 0.8 to 4.5 cm was used. At *ϕ* = 1.2, the cellularity affected the laminar flame speed calculation, with a notorious speed increase above a flame radius of 3.5 cm, as can be observed in Figure 8. Therefore, the radius window for the flame speed calculation was reduced to 0.8–3.5 cm. Similarly, at higher equivalence ratios, the radius window decreased due to increased flame instability. At equivalence ratios 1.3, 1.4, and 1.5, the radius windows used were 0.8–2.3, 0.7–2.1, and 0.7–1.9 cm, respectively. Considering these radius windows, the stretched laminar flame speed values were extrapolated to find the unstretched laminar flame speed, as shown in Figure 9.

**Figure 8:**
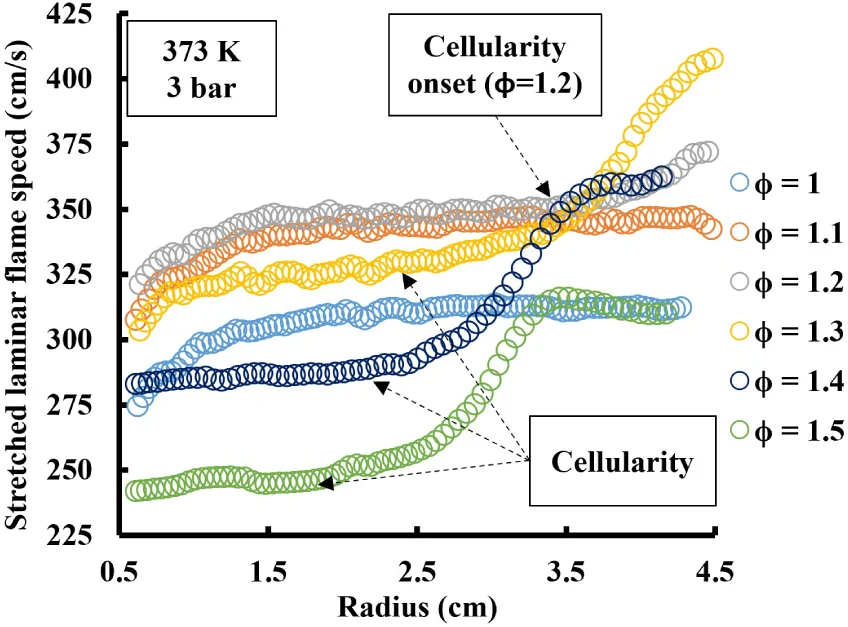
Stretched burned gas flame speed versus flame radius at 373 K and 3 bar.

**Figure 9:**
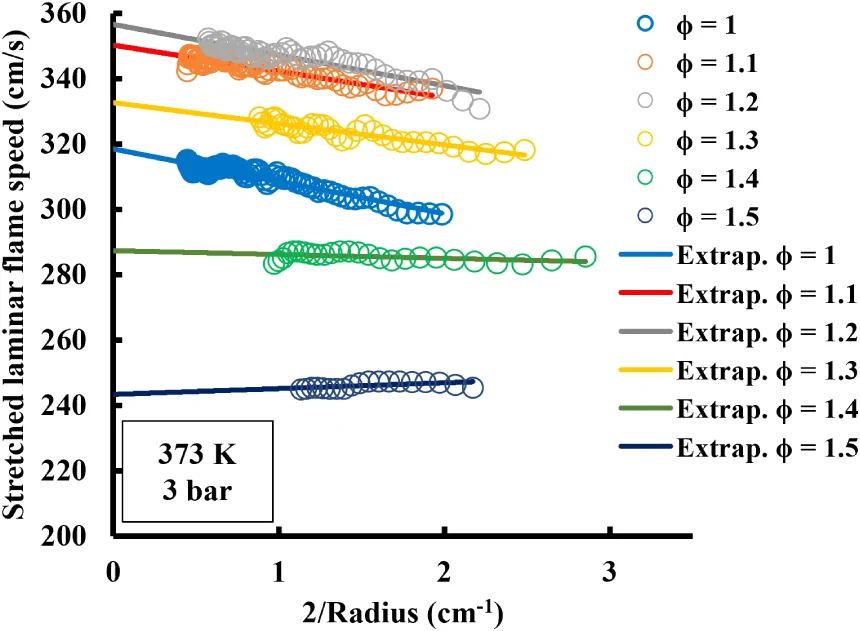
Stretched burned gas flame speed versus 2/*Radius* at 373 K and 3 bar.

Similar to the previous cases, at 5 bar and 373 K, cellularity affected the flame speed calculation, and the radius window had to be reduced for rich and stoichiometric mixtures. The stretched flame speed values at 5 bar and 373 K are shown in Figure 10. For equivalence ratios 0.7, 0.8, and 0.9, a radius window of 0.8–4.5 cm was used. At a stoichiometric equivalence ratio, cellularity started at 2.9 cm, and thereby, the radius window was adjusted to 0.8–2.9 cm. At equivalence ratios of 1.1, 1.2, 1.3, and 1.4, the radius windows were 0.8–2.4, 0.8–2, 0.6–1.5, and 0.5–1.1. Finally, with these radius windows, the stretched laminar flame speed values were extrapolated to eliminate the effect of stretch on the flame speed and calculate $S_{b}^{◦}$ , as indicated in Figure 11. The stretched burned gas flame speed values at 373 K, 5 bar, and *ϕ* = 1.2 are not shown in Figure 11 since they overlap with the values at *ϕ* = 1.1.

**Figure 10:**
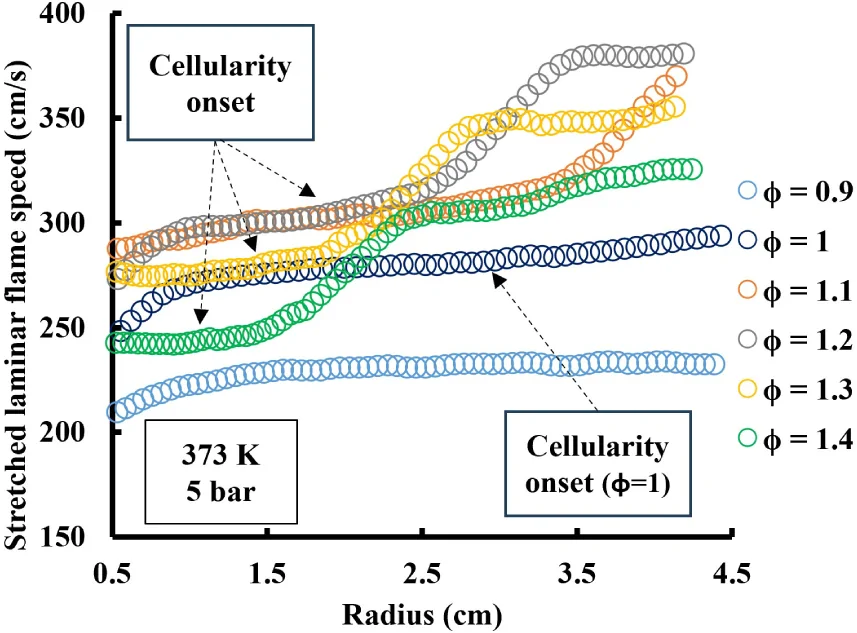
Stretched burned gas flame speed versus flame radius at 373 K and 5 bar.

**Figure 11:**
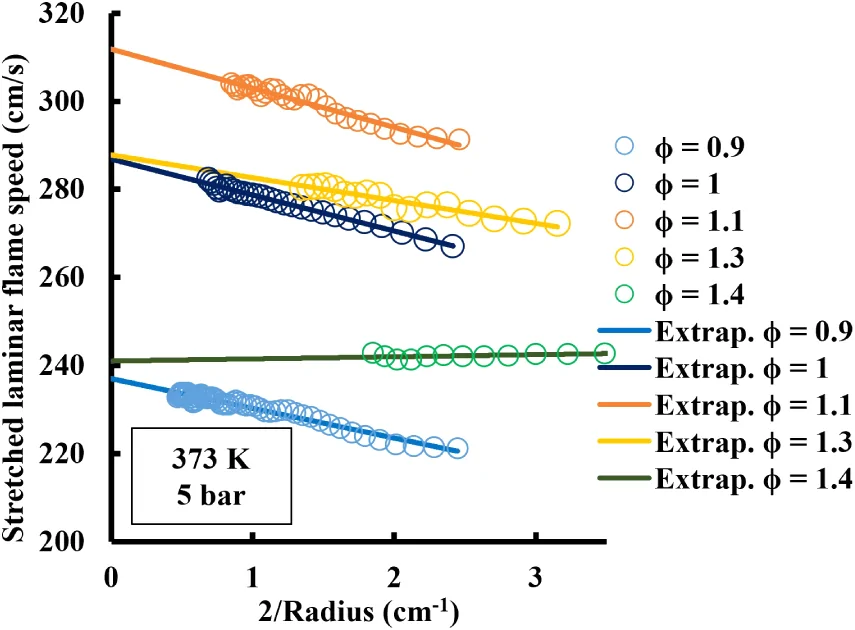
Stretched burned gas flame speed versus 2/*Radius* at 373 K and 5 bar.

At 423 K and 3 and 5 bar, the same procedure followed at 373 K was used, and similar radius windows were used for laminar flame speed extrapolation.

The measured values of unstretched laminar flame speed at 373, 423, and 473 K and 1 bar are shown in Figure 12. Generally, flame speed increases with increasing equivalence ratio for lean mixtures until *ϕ* = 1.2, then decreases for rich mixtures. The fuel/air mixtures at 373 and 423 K were ignitable at *ϕ* = 0.8. For mixtures at 473 K, ignition was achieved at *ϕ* = 0.7.

**Figure 12:**
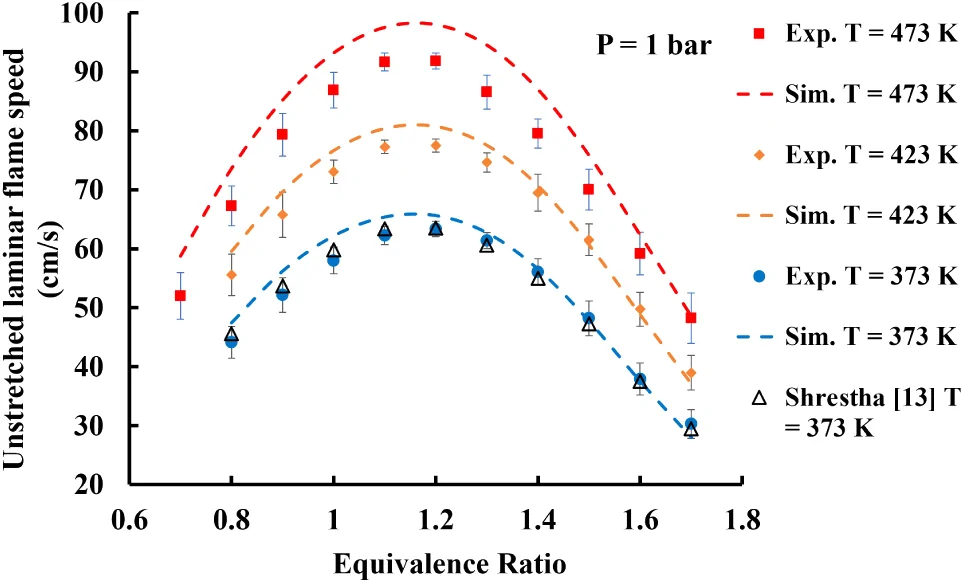
Experimental (solid icons) versus simulated values (dashed lines) of unstretched unburned gas laminar flame speed for *T* = 373, 423, and 473 K at 1 bar.

The maximum values of flame speed measured at 373, 423, and 473 K were 63.4, 77.5, and 91.8 cm/s, respectively, all at *ϕ* = 1.2, with total uncertainties of ±1.3, ±1.09, and ±1.37 cm/s. As a reference, the total uncertainties at 1 bar can be observed in Table 5. In all cases, the laminar flame speed increases with temperature, which increases molecular motion and particle kinetic energy, making collisions more frequent and energetic.^63^ As a result, these collisions exceed the activation energy, and chemical reactions occur faster. Therefore, the reaction rate is accelerated, and the flame speed increases.^64^ Moreover, temperature enhances molecular diffusion, thereby improving species transport.^65^

**Table 5:** Total Uncertainties in Laminar Flame Speed Measurements at Temperatures of 373, 423, and 473 K and a Pressure of 1 bar

	Total Uncertainty (cm/s)		
*ϕ*	*T* = 373 K, *P* = 1 bar	*T* = 423 K, *P* = 1 bar	*T* = 473 K, *P* = 1 bar
0.7	–	–	3.97
0.8	2.68	3.51	3.39
0.9	2.91	3.83	3.59
1.0	2.25	2.00	3.01
1.1	1.53	1.15	1.51
1.2	1.30	1.09	1.37
1.3	1.36	1.63	2.87
1.4	2.20	3.11	2.49
1.5	2.94	2.70	3.45
1.6	2.71	2.86	3.62
1.7	2.44	2.95	4.25

The experimental values at 373 K and 1 bar were compared with those reported by Shrestha et al.,^13^ and, as shown in Figure 12, excellent agreement is observed across all equivalence ratios. The highest difference was found at *ϕ* = 0.8, for which Shrestha et al.^13^ reported a laminar flame speed value of 45.61 cm/s, while in the present work, it was measured as 44.13 cm/s (a 3.2% difference). In the other equivalence ratios, the difference varies from 0 to 3%.

Comparing the numerical simulation with the experimental results, it is observed that the Jacobs et al.^14^ kinetic mechanism shows particularly good agreement at 373 K. At higher temperatures, the deviation increases in the lean and stoichiometric regions. However, the difference becomes smaller on the rich side. At 373 K, 1 bar, and equivalence ratios of 1.1, 1.2, and 1.3, the measured flame speed values are 62.3, 63.4, and 61.4 cm/s, while the model predicts 65.4, 65.6, and 62.7 cm/s, corresponding to a 5%, 3.55%, and 2.12% difference, respectively. At 473 K, 1 bar, and equivalence ratios of 1.1, 1.2, and 1.3, the measured flame speed values are 91.7, 91.8, and 86.6 cm/s, while the simulations predict 97.6, 98.0, and 94.6 cm/s, corresponding to a 6.45%, 6.74%, and 9.23% difference, respectively.

The effect of pressure on laminar flame speed can be observed in Figures 13 and 14. Generally, increasing pressure reduces flame speed, and the effect of pressure on laminar flame speed diminishes at higher pressures. At 373 K, the peak laminar flame speed values were found to be 49.8 cm/s at 3 bar and *ϕ* = 1.2, and 43.2 cm/s at 5 bar and *ϕ* = 1.1, with total uncertainties of ±0.9, and ±0.6 cm/s, respectively.

**Figure 13:**
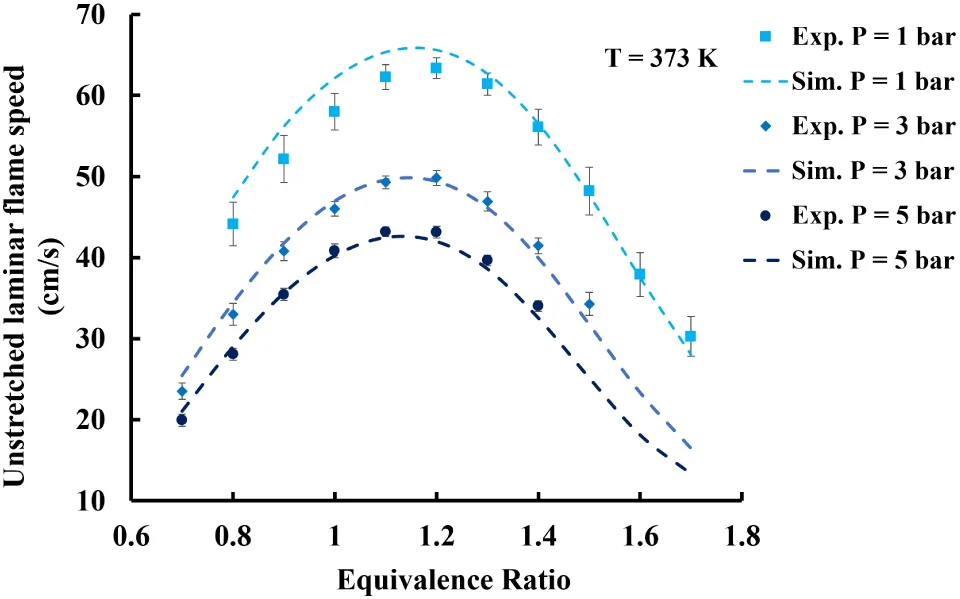
Experimental (solid icons) versus simulated values (dashed lines) of unstretched unburned gas laminar flame speed for 1, 3, and 5 bar at 373 K.

**Figure 14:**
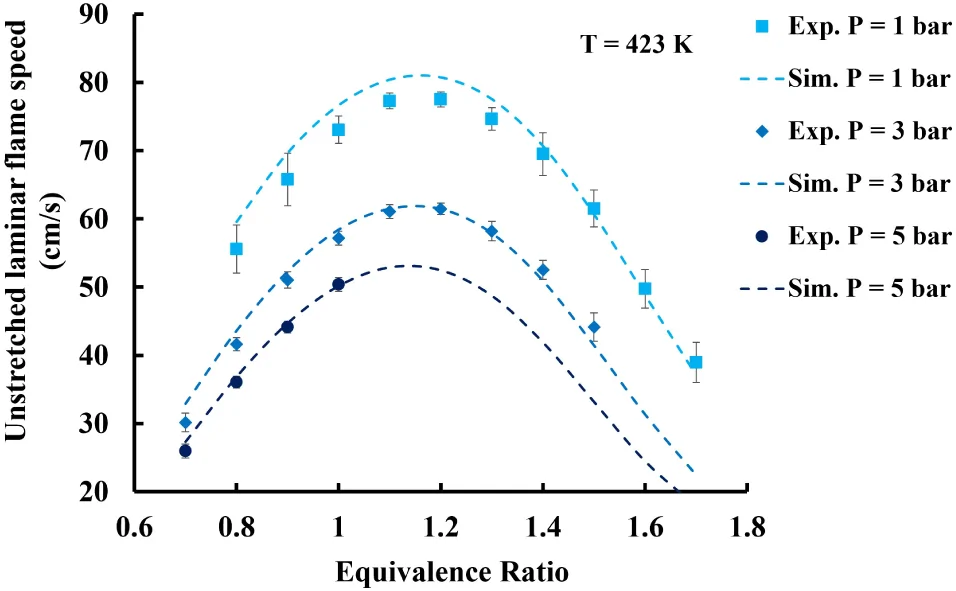
Experimental (solid icons) versus simulated values (dashed lines) of unstretched unburned gas laminar flame speed for 1, 3, and 5 bar at 423 K.

In addition, at 423 K and 3 bar, the maximum flame speed was 61.5 ± 0.83 cm/s at *ϕ* = 1.2. At 3 bar and temperatures of 373 and 423 K, the laminar flame speed at equivalence ratios 1.6 and 1.7 is not shown due to highly unstable and wrinkled flames, for which it was not possible to calculate the laminar flame speed. Similarly, at 373 K and 5 bar, intense cellularity was observed at equivalence ratios of 1.5, 1.6, and 1.7, and therefore, the laminar flame speed was not calculated for these conditions. At 423 K and 5 bar, the laminar flame speed is measured only up to *ϕ* = 1 due to fuel autoignition and elevated pressure gradients in the vessel. Thus, the maximum flame speed at 423 K and 5 bar was not determined, since it usually occurs in the range of *ϕ* = 1.1–1.2. At 423 K, 5 bar, and *ϕ* = 1, the measured flame speed was 50.4 ± 1.01 cm/s. Experimental tests at 473 K and higher pressures (3 and 5 bar) were not carried out to avoid damage to the quartz windows, given the autoignition of the fuel at 423 K and 5 bar.

Laminar flame speed decreases with increasing pressure because three-body reactions (termination reactions) are favored over two-body reactions. As a result, a retarding effect is generated on the overall advance of the reaction. Furthermore, a higher initial pressure also increases the density of unburned gas, *ρ*_*u*_, which reduces the flame speed according to eq 9.^52,64^

At higher pressures (3 and 5 bar), the Jacobs et al.^14^ mechanism accurately predicts the experimental flame speeds at equivalence ratios from 0.8 to 1.4, with underpredictions higher than 6% at an equivalence ratio of 1.5.

The Markstein length values extracted from the laminar flame speed extrapolations are presented in Figures 15, 16, and 17. At all conditions, a clear tendency of reduction of Markstein length with increasing equivalence ratio can be observed. At 373 K, 1 bar, and equivalence ratios of 0.8, 1, and 1.7, the Markstein length decreases from 1.14 ± 0.054 to 0.904 ± 0.0071 and −0.73 ± 0.015 mm, respectively. This reduction in Markstein length with increasing equivalence ratio can be explained based on the effect of equivalence ratio on the preferential diffusion of the mixture, and on the effective Lewis number.^29^ Several studies on Markstein length^36,59,66,67^ showed that it depends on the effective Lewis number *Le*_*eff*_, density ratio *σ*, Zel’dovich number *Ze,* and preheat flame thickness *δ*_*l*_ (defined in eq 20 as a thermal diffusivity to flame speed ratio) and can be expressed as eq 19:^29,67^

19 $$L_{b}=\delta_{l}\times \left[f_{1}\left(\sigma \right)+Ze\times \left(Le_{eff}-1\right)\times f_{2}\left(\sigma \right)\right]$$

20 $$\delta_{l}=\frac{\alpha }{S_{u}^{◦}}$$

where the functions *f*_1_(*σ*) and *f*_2_(*σ*) depend on the density ratio and have positive values. The first term in eq 19 is always positive and becomes relevant in mixtures with *Le*_eff_ = 1 (equidiffusion). On the contrary, the second term represents the thermodiffusive effect on Markstein length for mixtures with preferential diffusion (nonequidiffusion) and becomes negative when *Le*_*eff*_ < 1. As will be discussed in Section 3.4, the effective Lewis number of the mixture is significantly higher than one for lean mixtures and decreases with increasing equivalence ratios, as shown in Figure 28, since the mass diffusivity of DMM is lower than that of oxygen. Consequently, as the effective Lewis number of the mixture decreases, the thermodiffusive component *Ze* × (*Le*_*eff*_ – 1) × *f*_2_(*σ*) decreases, which generates a reduction in *L*_*b*_. For this reason, the decrease in Markstein length with increasing equivalence ratios coincides with the reduction in effective Lewis number at higher equivalence ratios. This dependence of Markstein length on the effective Lewis number arises from the effect of preferential diffusion on the flame response to stretch.

**Figure 15:**
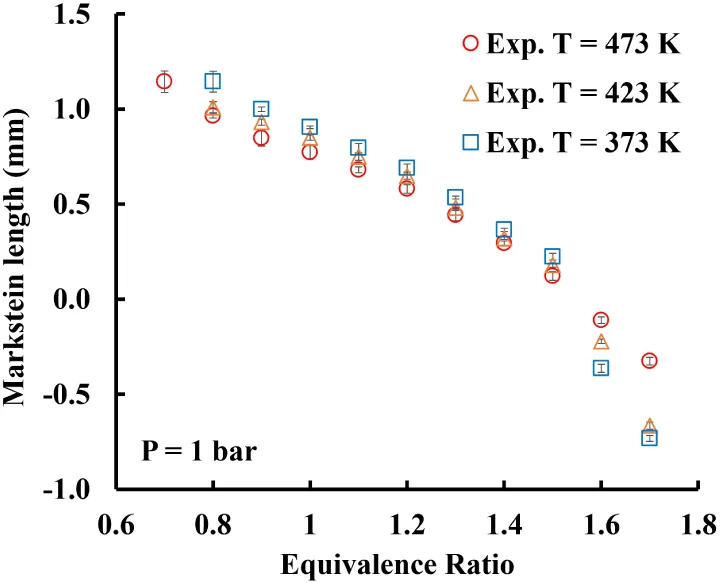
Markstein length *L_b_* versus equivalence ratio for DMM flames at 1 bar and 373, 423, and 473 K.

**Figure 16:**
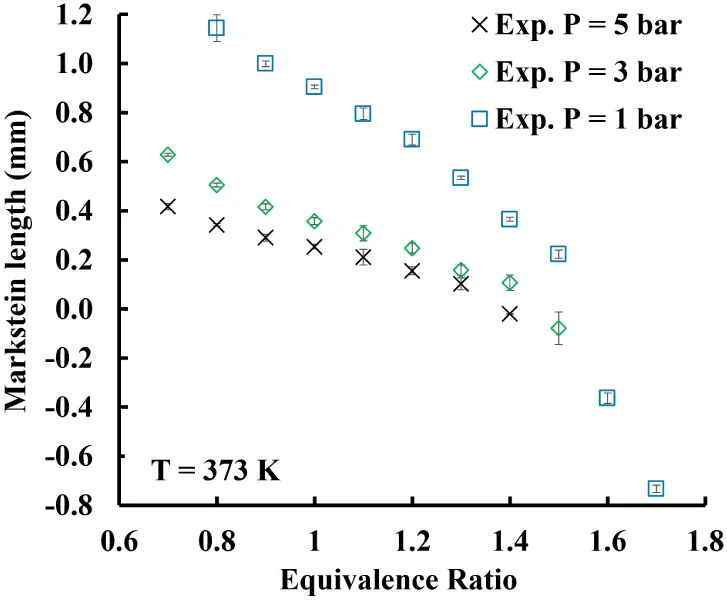
Markstein length *L_b_* versus equivalence ratio for DMM flames at 373 K and 1, 3, and 5 bar.

**Figure 17:**
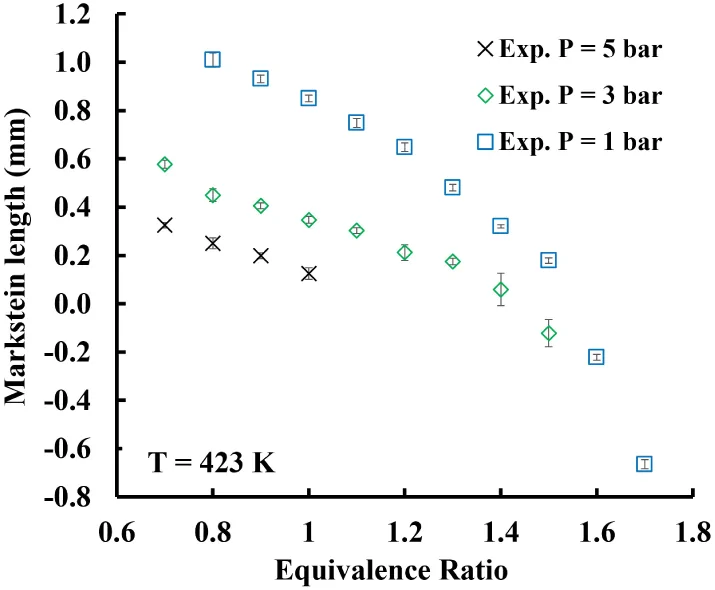
Markstein length *L_b_* versus equivalence ratio for DMM flames at 423 K and 1, 3, and 5 bar.

In a spherically expanding flame (where the stretch rate is always positive), curvature affects the mass and heat transport into and from the reaction zone. When the effective Lewis number is different from one, the different diffusion velocities can produce a local increase or decrease in the deficient reactant concentration. As a result, the local flame speed can increase or decrease depending on *Le*_*eff*_, and the flame becomes sensitive to stretch. As mentioned in Section 2.4, if *Le*_*eff*_ > 1, heat diffusion is faster than mass diffusion of the deficient reactant, and thereby, a local depletion of the limiting reactant occurs in the reaction zone of the stretch flame, which reduces the burning speed at that point. Consequently, the Markstein length becomes positive (reduction of the flame speed with increasing stretch), resulting in a stabilizing effect on flame perturbations. On the contrary, when *Le*_*eff*_ < 1, the opposite effect happens. The deficient reactant diffuses faster than heat, causing a local increase in the concentration and flame speed. Therefore, the Markstein length becomes negative (increasing flame speed with increasing stretch rate), contributing to the amplification of perturbations and promoting flame instability.^52,57,59,67^

The effect of temperature on Markstein length (Figure 15) was found to be small (relative to the effect of equivalence ratio and pressure). A weak reduction of Markstein length with increasing temperature was observed from *ϕ* = 0.8 to 1.5, indicating that the flame becomes slightly more unstable at higher temperatures. At 1 bar, *ϕ* = 0.8, and 373, 423, and 473 K, the Markstein length values were 1.14 ± 0.054, 1.01 ± 0.029, and 0.965 ± 0.0127 mm, respectively. A similar tendency of decreasing Markstein length with increasing temperature was reported by Di et al.^68^ for diethyl ether flames and Sun et al.^30^ for DMM/ammonia mixtures. This reduction of Markstein length with increasing temperature arises from the small decrement of effective Lewis number, Zel’dovich number, and preheat flame thickness with increasing temperature, as can be observed in Figure 28 and Figure 18 a and c.

**Figure 18:**
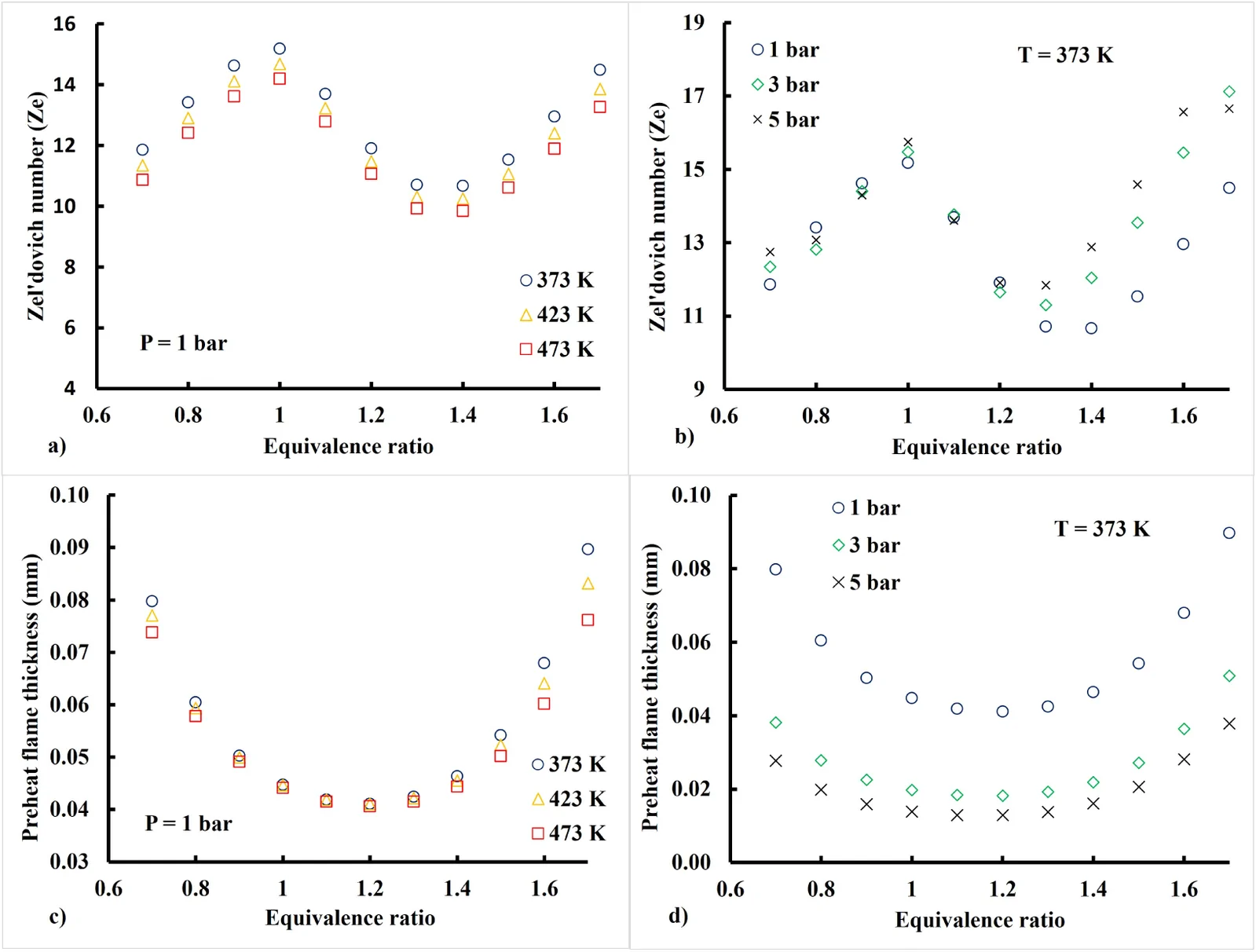
Effect of temperature, pressure, and equivalence ratio on Zel’dovich number and preheat flame thickness.

It is interesting to note that at 1 bar, and temperatures of 373, 423, and 473 K, the Markstein length becomes negative above *ϕ* = 1.5, and a significant reduction in *L*_*b*_ at *ϕ* = 1.6 and 1.7 can be observed. This indicates that the thermodiffusive term is negative and contributes to a destabilizing response of the flame to stretch. In addition, at ϕ = 1.6 and 1.7, the Markstein length deviates from the behavior observed at lower equivalence ratios. Specifically, the Markstein length does not follow the monotonic reduction with increasing equivalence ratio and the weak decrease with increasing temperature. A similar behavior was reported by Galmiche et al.^69^ for iso-octane flames at 1 bar, temperatures between 323 and 473 K, and high equivalence ratios (1.5–1.6). Even though a mechanistic explanation was not provided by the authors, this behavior coincides with the region of negative Markstein length and increased thermodiffusive instability, which indicates that preferential diffusion alters the flame behavior and increases its sensitivity to stretch.

The effect of pressure on Markstein length can be observed in Figures 16 (373 K) and 17 (423 K). A notorious reduction of *L*_*b*_ with increasing pressure was measured at both 373 and 423 K and particularly in the lean region. At 373 K, *ϕ* = 0.9 at 1, 3, and 5 bar, the Markstein length values were 1 ± 0.0109, 0.417 ± 0.0121, and 0.29 ± 0.0144 mm, respectively. Similarly, at 423 K, *ϕ* = 0.9, and 1, 3, and 5 bar, the Markstein length values were 0.931 ± 0.0161, 0.405 ± 0.012, and 0.199 ± 0.0125 mm, respectively. The tendency of the Markstein length to decrease with increasing pressure was previously reported by Di et al.^68^ for diethyl ether flames, Sun et al.^30^ for DMM/ammonia mixtures, Okafor et al.^70^ for *H*_2_–*CH*_4_ flames, and Li et al.^71^ for 2,5-dimethylfuran flames.

As discussed in Section 3.4, the increase in pressure does not affect the effective Lewis number, since both mass and heat diffusivities have the same inverse dependence on pressure. The main effect of pressure occurs on the preheat flame thickness that decreases significantly with increasing pressure, as shown in Figure 18d. Since the Markstein length is scaled by the preheat flame thickness, it decreases with increasing pressure.

### 3.2 Comparison with Kinetic Mechanisms Available in the Literature

The comparison of numerical and experimental laminar flame speed values is shown in Figures 19 and 20.

**Figure 19:**
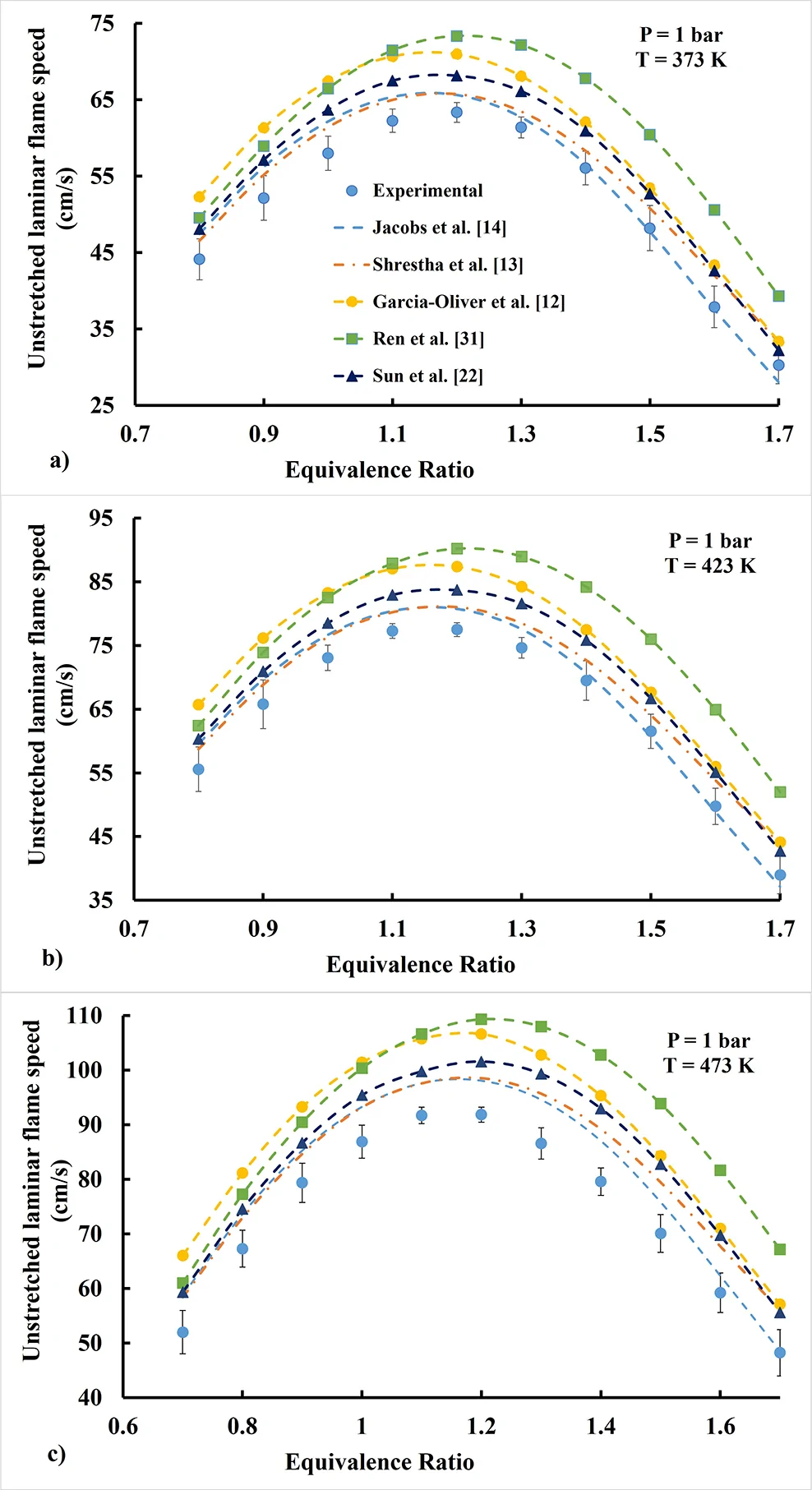
Comparison of experimental laminar flame speed measurements with numerical results from several kinetic mechanisms at 1 bar and 373, 423, and 473 K.

**Figure 20:**
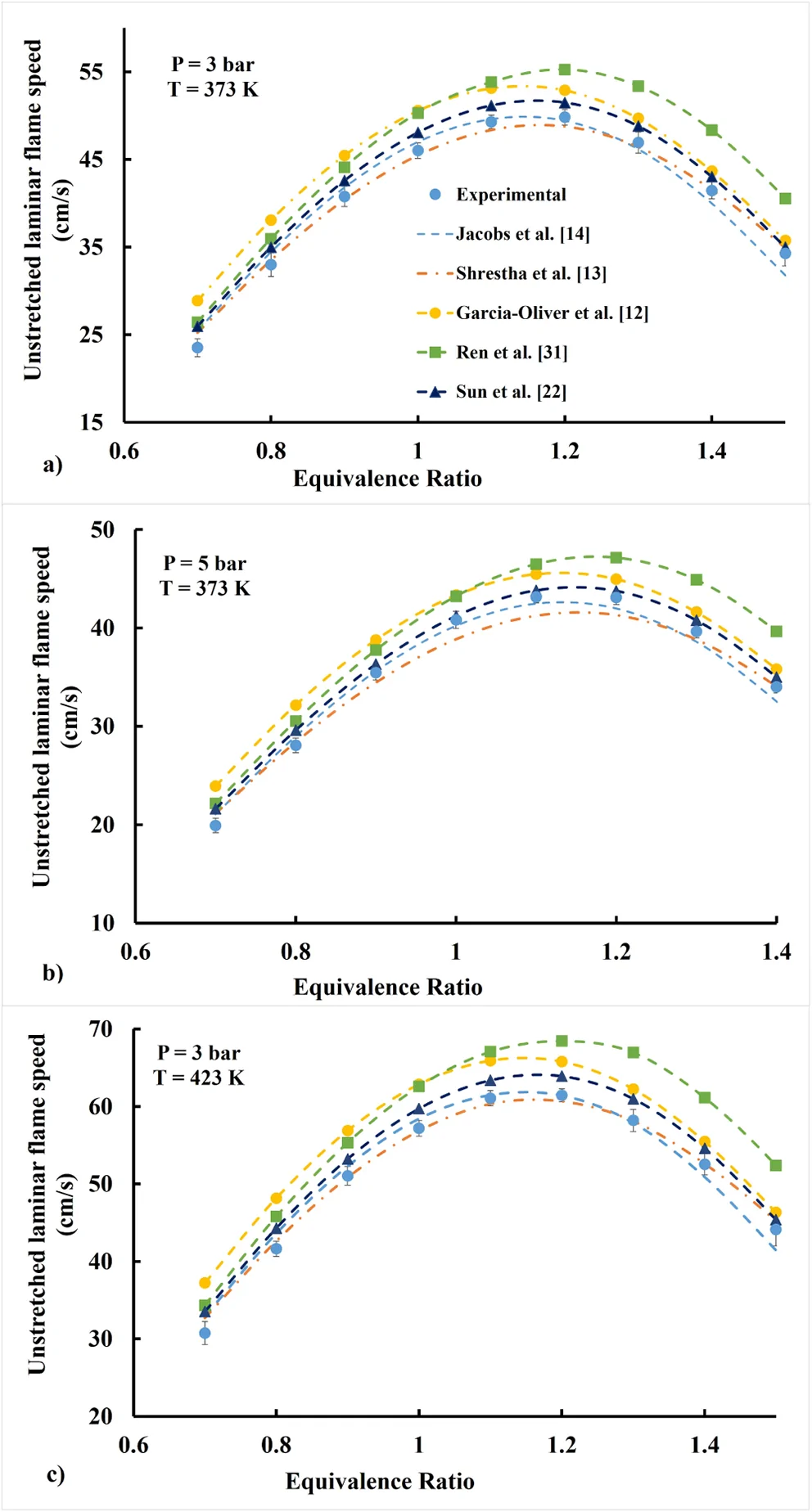
Comparison of experimental laminar flame speed measurements with numerical results from several kinetic mechanisms at 3 and 5 bar at 373 K and 3 bar and 423 K.

At 1 bar and 373 K, in the lean region, the mechanisms developed by Jacobs et al.^14^ and Shrestha et al.^13^ show higher laminar flame speed values than the experimental results. In addition, at rich equivalence ratios, the mechanism by Shrestha et al.^13^ overestimates the experimental results, while the mechanism from Jacobs et al.^14^ shows good agreement. The GarcÍa-Oliver et al.^12^ mechanism yields the highest values of all under lean conditions. Nonetheless, the model’s predictions improve modestly at higher equivalence ratios. The Ren et al.^31^ mechanism substantially overestimates the laminar flame speed, particularly under rich conditions, while the Sun et al.^22^ mechanism overpredicts, by a smaller margin, in the lean region but becomes less precise at high equivalence ratios.

As a reference, at 373 K, 1 bar, and *ϕ* = 1, the experimental laminar flame speed value is 58.0 cm/s, while the Jacobs et al.^14^ and Shrestha et al.^13^ mechanisms predict a laminar flame speed value of 62.2 and 61.4 cm/s, corresponding to a 7.2% and 5.9% difference. The mechanisms from García-Oliver et al.,^12^ Ren et al.,^31^ and Sun et al.^22^ predict a value of 67.4, 66.5, and 63.7 cm/s, corresponding to a 16.3%, 14.6%, and 9.8% difference, respectively.

At higher temperatures (423 and 473 K) and 1 bar pressure, the same trends are repeated, with both the Jacobs et al.^14^ and Shrestha et al.^13^ mechanisms showing better approximation in the lean region, while on the rich side, the Jacobs et al.^14^ mechanism shows better agreement.

At 373 K and 3 bar, experimental flame speed values are lower than those at 1 bar, and the Jacobs et al.,^14^ Shrestha et al.,^13^ and Sun et al.^22^ mechanisms all show good agreement in the lean, stoichiometric, and slightly rich regions (*ϕ* = 0.8–1.4). At a higher equivalence ratio (*ϕ* = 1.5), the Jacobs et al.^14^ mechanism underpredicts the experimental values, but the Shrestha et al.,^13^ García-Oliver et al.,^12^ and Sun et al.^22^ mechanisms all produce results closer to the experimental values. The Ren et al.^31^ mechanism overpredicts the experimental results at all equivalence ratios.

At 373 K and 5 bar, the Jacobs et al.,^14^ Shrestha et al.,^13^ and Sun et al.^22^ mechanisms show particularly good agreement with the experimental results at lean, stoichiometric, and slightly rich conditions (*ϕ* = 0.8–1.4). The García-Oliver et al.^12^ mechanism overestimates the experimental measurements, especially in the lean and stoichiometric regions. The Ren et al.^31^ mechanism overestimates the measured values at all equivalence ratios and deviates more significantly at richer conditions.

At 423 K and 3 bar, the Jacobs et al.,^14^ Shrestha et al.,^13^ and Sun et al.^22^ mechanisms show notably good agreement in the lean and slightly rich regions (*ϕ* = 0.9–1.4). However, at *ϕ* = 1.5, the Jacobs et al.^14^ mechanism underestimates the experimental results while the Shrestha et al.,^13^ Sun et al.,^22^ and García-Oliver et al.^12^ mechanisms produce better estimations. As in the previous cases, the Ren et al.^31^ mechanism deviates from the experimental results by a wide margin.

A summary of the average error of the simulated laminar flame speed values relative to the experimental results can be observed in Table 6. At 1 bar and 373, 423, and 473 K, the mechanism from Jacobs et al.^14^ shows, on average, 4.3%, 4%, and 7.6% errors, respectively, relative to the experimental laminar flame speed results. Thus, this mechanism is the most accurate at a low pressure. At 3 bar and temperatures of 373 and 423 K, the mechanism from Shrestha et al.^13^ has relative errors of 2.0% and 1.7%, respectively, and thus, it is the most precise under these conditions. At 373 K and 5 bar, the Jacobs et al.^14^ and Shrestha et al.^13^ mechanisms have average errors of 2.7% and 3.2%, respectively. Therefore, the Jacobs et al.^14^ mechanism is slightly more precise.

**Table 6:** Average Error of the Simulated Laminar Flame Speeds with Each Kinetic Mechanism at the Studied Conditions

			Average absolute error				
Temperature (K)	Pressure (bar)	Range of equivalence ratios	Jacobs et al.^14^	García-Oliver et al.^12^	Shrestha et al.^13^	Ren et al.^31^	Sun et al.^22^
373	1	0.8–1.7	4.3%	13.5%	5.9%	19.8%	8.9%
373	3	0.7–1.5	3.4%	9.9%	2.0%	12.0%	4.7%
373	5	0.7–1.4	2.7%	8.8%	3.2%	9.9%	3.3%
423	1	0.8–1.7	4.0%	13.3%	5.7%	19.5%	8.6%
423	3	0.7–1.5	3.0%	10.1%	1.7%	12.3%	4.8%
473	1	0.7–1.7	7.6%	19.1%	10.5%	23.8%	13.3%

### 3.3 Sensitivity and Reaction Pathway Analysis

In order to identify the main chemical reactions affecting the laminar flame speed, a sensitivity analysis is carried out at equivalence ratios of 0.7, 1, and 1.7 under different temperatures and pressures. The top 16 reactions are shown in Figures 21, 22, and 23. From bottom to top, the reactions with positive and negative coefficients are displayed in an alternating order. The reactions with the highest absolute sensitivity coefficients are located closer to the horizontal axis.

**Figure 21:**
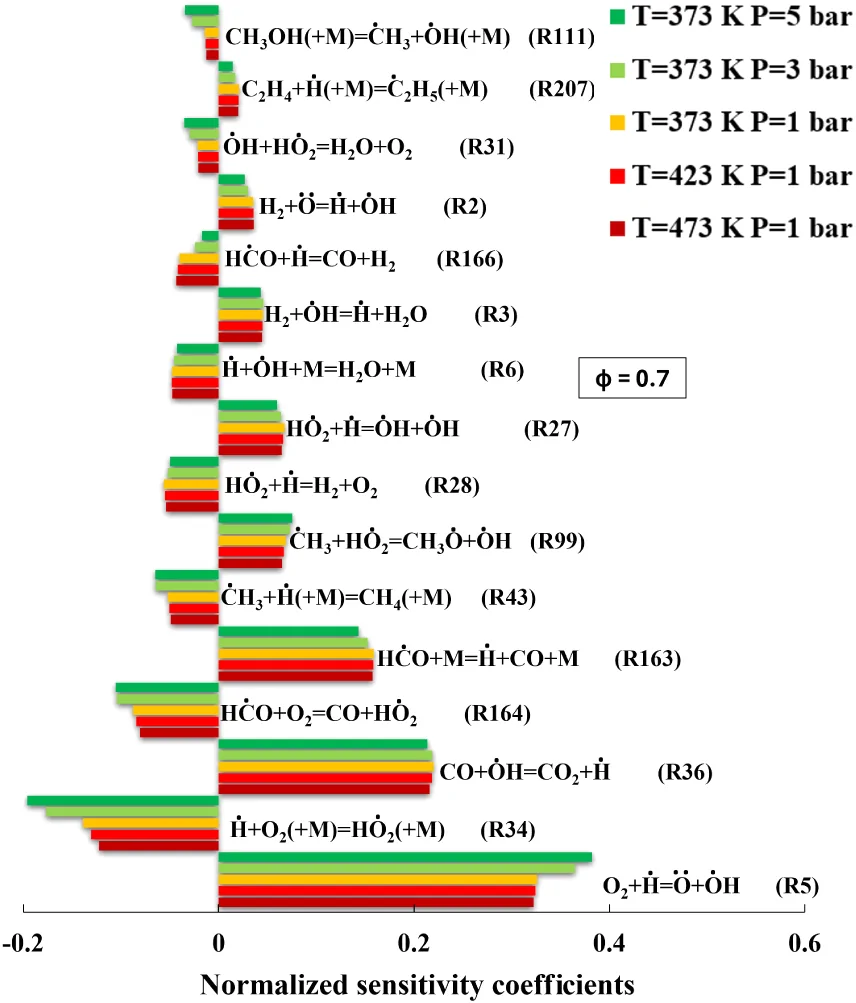
Flame speed sensitivity coefficients for DMM/air flames at *ϕ* = 0.7 and several temperatures and pressures.

**Figure 22:**
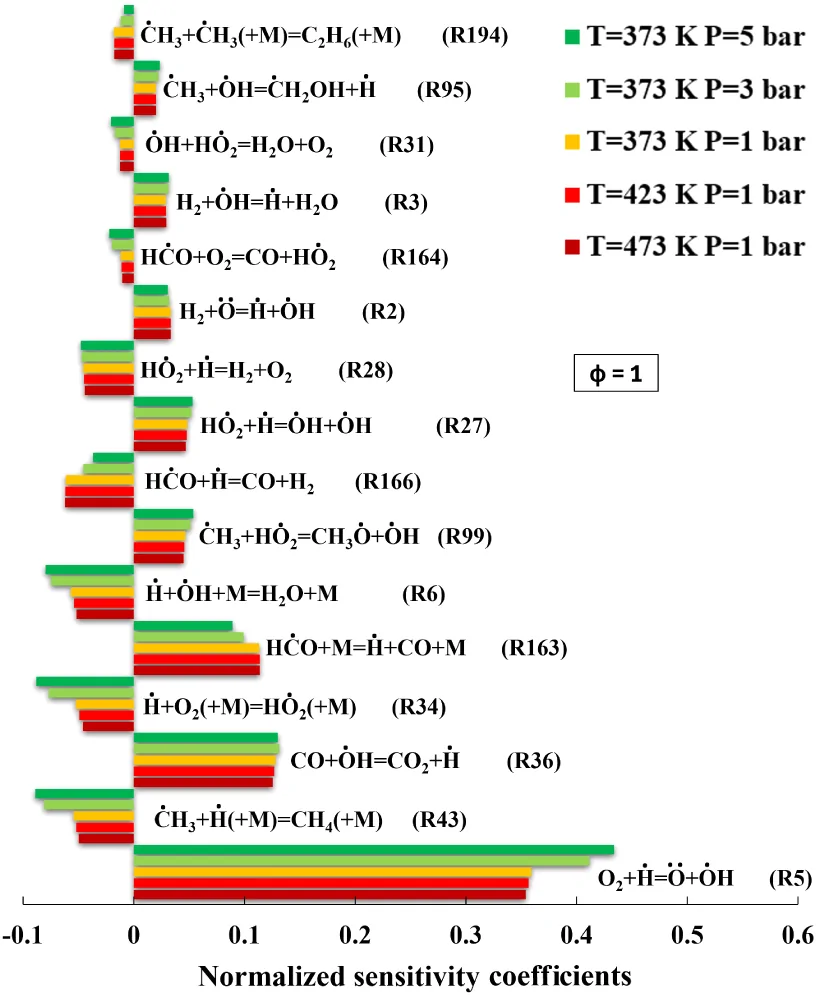
Flame speed sensitivity coefficients for DMM/air flames at *ϕ* = 1 and several temperatures and pressures.

**Figure 23:**
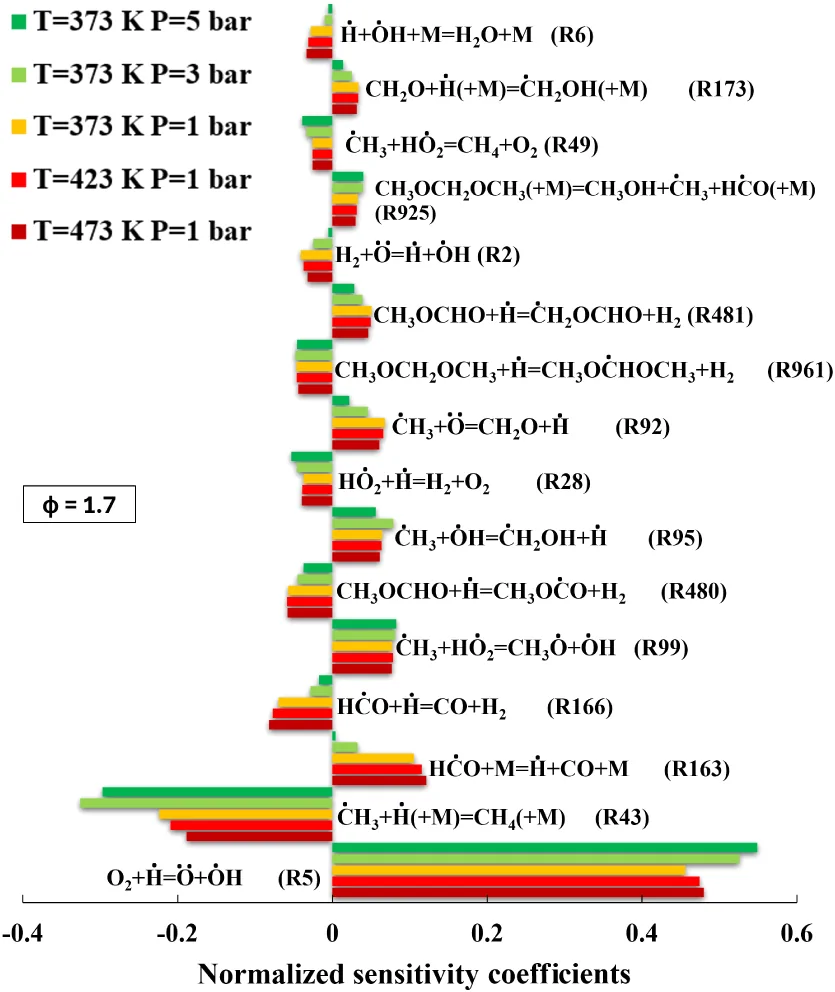
Flame speed sensitivity coefficients for DMM/air flames at *ϕ* = 1.7 and several temperatures and pressures.

The reactions with positive normalized coefficients promote reactivity and boost laminar flame speed while the reactions with negative coefficients reduce reactivity and decrease laminar flame speed. Generally, chain-branching reactions increase reactivity because they produce highly important reactive radicals like hydroxyl radical ȮH or hydrogen radical Ḣ that participate in crucial combustion reactions. In contrast, chain-terminating reactions reduce reactivity because they consume important radicals to give other combustion products.^72^

The flame speed sensitivity coefficients at an equivalence ratio of 0.7 are presented in Figure 21. As can be observed, most of the important reactions belong to the H/O radical pool. The chain-branching reaction R5 O_2_ + Ḣ = Ö + ȮH has the highest sensitivity coefficients under all conditions and thus is the most influential on laminar flame speed. It generates 2 reactive radicals: monatomic oxygen Ö and hydroxyl radical ȮH, both of which are potent oxidizing species that contribute to fuel breakdown. Other reactions that produce ȮH, Ḣ, and methoxy radicals CH_3_Ȯ like reactions R36 CO + ȮH = CO_2_ + Ḣ, R163 HĊO + *M* = Ḣ + CO + *M*, R99 ĊH_3_ + HȮ_2_ = CH_3_Ȯ + ȮH, and R27 HȮ_2_ + Ḣ = ȮH + ȮH also increase reactivity.

The chain-terminating reaction R34 Ḣ + O_2_ + *M* = HȮ_2_ + *M* directly competes with reaction R5, consuming Ḣ from the radical pool to form the less reactive radical hydroperoxyl HȮ_2_ and thus reduces reactivity.^40,72^ This reaction has the most negative coefficients of all analyzed reactions at *ϕ* = 0.7 and thus is the one that hinders the laminar flame speed the most.

The sensitivity coefficients do not change significantly with temperature. Nonetheless, at higher pressures, three-body reactions such as R34 and R43 are enhanced relative to two-body reactions because the third-body concentration increases, as does the probability of reactant collisions with third bodies, thereby allowing the reactions to occur. As a result, these reactions are accelerated, consuming radicals and reducing the flame speed.

Even though reaction R5 O_2_ + Ḣ = Ö + ȮH is bimolecular (no third bodies involved), its sensitivity coefficient significantly increases with pressure, from 0.326 at 373 K and 1 bar to 0.365 and 0.382 at 3 and 5 bar, respectively. This increase implies that even at higher pressures, where the radical pool is depleted, the reaction continues to significantly influence laminar flame speed.

Finally, the reaction R36 CO + ȮH = CO_2_ + Ḣ is key in the *CO* oxidation mechanism and heat release, which increases reactivity.^72^

Fuel-specific reactions were not found to be substantially influential on laminar flame speed at *ϕ* = 0.7. A similar observation was pointed out by Jacobs et al.^14^ The main reaction involving DMM is R961 CH_3_OCH_2_OCH_3_ + Ḣ = CH_3_OĊHOCH_3_ + H_2_ with a normalized coefficient of −0.012 at 373 K, 5 bar, and *ϕ* = 0.7. However, reactions involving radicals that are the products of fuel decomposition, such as formyl radical HĊO (R164 HĊO + *O*_2_ = CO + HȮ_2_ and R163 HĊO + *M* = Ḣ + CO + *M*), and methyl radical (R43 ĊH_3_ + Ḣ + *M* = CH_4_ + *M* and R99 ĊH_3_ + HȮ_2_ = CH_3_Ȯ + ȮH), have higher sensitivity coefficients.

Jacobs et al.^14^ identified reactions R5 O_2_ + Ḣ = Ö + ȮH, R36 CO + ȮH = CO_2_ + Ḣ, and R163 HĊO + *M* = Ḣ + CO + *M* as key for the DMM laminar flame speed at 328 K and 1 atm. In addition, Duva and Toulson^40^ pointed out that the chain-terminating reaction R34 Ḣ + O_2_ + *M* = HȮ_2_ + *M* largely inhibits laminar flame speed in *H*_2_/air flames, *H*_2_ *O* being the main third body responsible for this reaction in diluted mixtures with exhaust gas recirculation (EGR).

For DMM flames with an equivalence ratio of 1, the sensitivity coefficients can be observed in Figure 22. The top 14 reactions at *ϕ* = 0.7 also appear at *ϕ* = 1, with some variations in the coefficient magnitude and the relative order of importance. It can be noted that reaction R5 O_2_ + Ḣ = Ö + ȮH continues to be the most influential. The H/O chemistry is still highly relevant at *ϕ* = 1; however, the importance of fuel chemistry increases, since at higher equivalence ratios, *O*_2_ is less abundant and the fuel fragment concentration increases. In addition, the absolute sensitivity coefficients of reactions involving the hydroperoxyl radical HȮ_2_ (R99 ĊH_3_ + HȮ_2_ = CH_3_Ȯ + ȮH, R28 HȮ_2_ + Ḣ = H_2_ + O_2_, and R27 HȮ_2_ + Ḣ = ȮH + ȮH) and molecular oxygen *O*_2_ (R34 Ḣ + O_2_ + *M* = HȮ_2_ + *M*, R164 HĊO + *O*_2_ = CO + HȮ_2_) decrease, and thus, they become less influential. On the contrary, reactions R43 ĊH_3_ + Ḣ + *M* = CH_4_ + *M*, R6 Ḣ + ȮH + *M* = H_2_O + *M*, and R166 HĊO + Ḣ = CO + H_2_ become more influential.

The reactions involving methanol R111 CH_3_OH + *M* = ĊH_3_ + ȮH + *M* and ethylene R207 C_2_H_4_ + Ḣ + *M* = C_2_H_5_ + *M* previously included in the plot at *ϕ* = 0.7 disappear at *ϕ* = 1, and 2 new reactions involving methyl radicals R194 ĊH_3_ + ĊH_3_ + *M* = C_2_H_6_ + *M* and R95 ĊH_3_ + ȮH = ĊH_2_OH + Ḣ are included.

In Figure 23, the sensitivity coefficients for DMM flames at *ϕ* = 1.7 are presented. As in the previous cases, reaction R5 O_2_ + Ḣ = Ö + ȮH remains the most influential reaction, with the highest sensitivity coefficients. In addition, its sensitivity coefficients increase under all conditions.

Under rich conditions, *O*_2_ is scarce while the fuel concentration is high. Therefore, reactions involving the products of fuel decomposition become increasingly influential on the chemistry of the flame. Not surprisingly, the reactions involving *O*_2_ (R34 Ḣ + O_2_ + *M* = HȮ_2_ + *M* and R164 HĊO + *O*_2_ = CO + HȮ_2_) do not make it to the top 16 and do not appear in the plot at *ϕ* = 1.7. Moreover, reaction R36 that describes the complete oxidation of carbon monoxide to form carbon dioxide CO + ȮH = CO_2_ + Ḣ is not present in the top 16 reactions. Other reactions that are not part of the top 16 at *ϕ* = 1.7 are R27 HȮ_2_ + Ḣ = ȮH + ȮH, R3 H_2_ + ȮH = Ḣ + H_2_O, R31 ȮH + HȮ_2_ = H_2_O + O_2_, and R194 ĊH_3_ + ĊH_3_ + *M* = C_2_H_6_ + *M*

At *ϕ* = 1.7, several new reactions are included in the top 16 and most of them involve products of fuel breakdown. For instance, the top 16 include reactions involving methyl formate (R480 CH_3_OCHO + Ḣ = CH_3_OĊO + H_2_ and R481 CH_3_OCHO + Ḣ = ĊH_2_OCHO + H_2_), methyl radical (R92 ĊH_3_ + Ö = CH_2_O + Ḣ and R49 ĊH_3_ + HȮ_2_ = CH_4_ + O_2_), and formaldehyde (R173 CH_2_O + Ḣ + *M* = ĊH_2_OH + *M*). In addition, for the first time, reactions directly involving DMM can be observed in the top 16, such as R925 CH_3_OCH_2_OCH_3_ + *M* = CH_3_OH + ĊH_3_ + HĊO + *M* and the secondary hydrogen abstraction R961 CH_3_OCH_2_OCH_3_ + Ḣ = CH_3_OĊHOCH_3_ + H_2_.

It is worth noting that at *ϕ* = 0.7 and 1, 8 out of the 16 reactions include a carbon-containing species, while at *ϕ* = 1.7, the number of reactions involving species that contain carbon atoms increases to 12.

A reaction pathway analysis was carried out for DMM at 373 K, 1 bar, an equivalence ratio of 1, and 20% fuel consumption. The 20% DMM conversion level was chosen since at this stage, a radical pool is already established, while the DMM concentration in the mixture remains high. In addition, secondary intermediate species still do not dominate the global chemistry of the system. Therefore, the reaction pathway is representative of the DMM-oxidation process. A similar approach was used by Jacobs et al.^14^ and Li et al.^73^ for DMM reaction pathway analyses.

In Figure 24, the reaction pathway for the secondary DMM radical CH_3_OĊHOCH_3_ is presented, while in Figure 25, the reaction pathway for the primary DMM radical CH_3_OCH_2_OĊH_2_ can be observed. The numbers in blue indicate the relative rate of production of each species.

**Figure 24:**
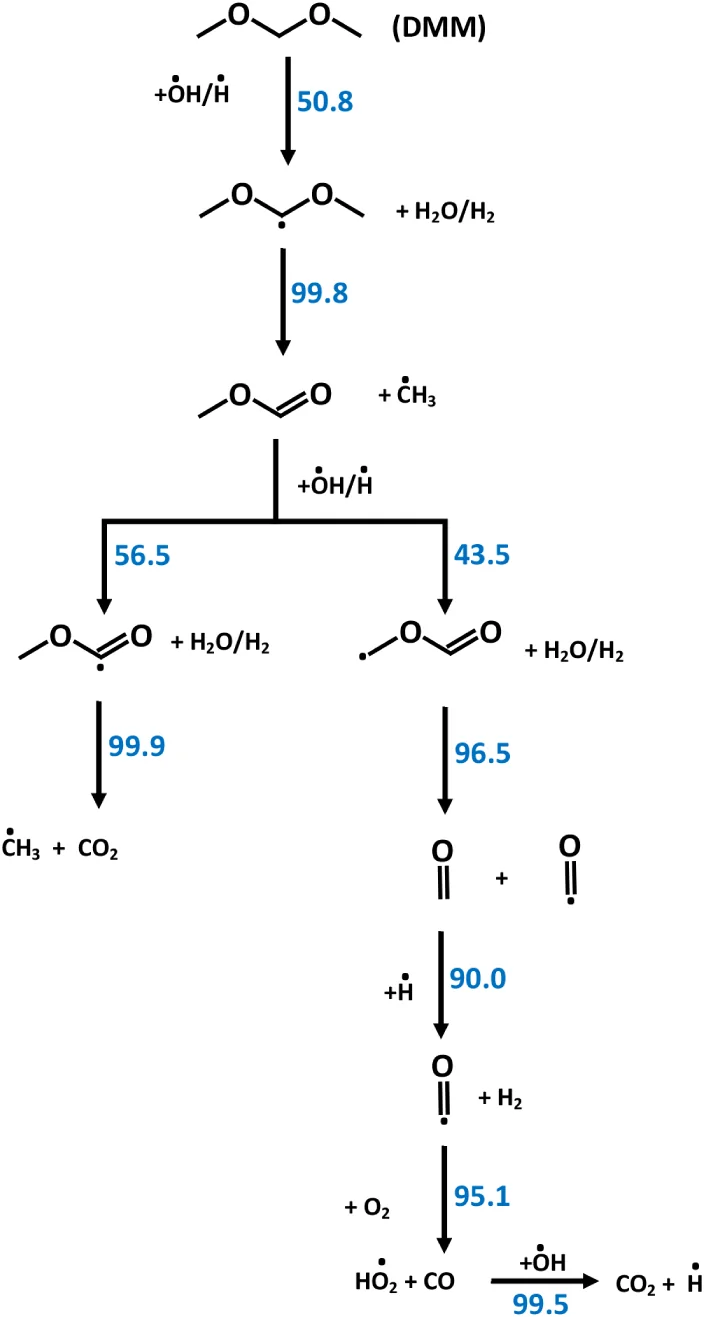
Reaction pathway for secondary DMM radical formation at 373 K, *ϕ* = 1, 1 bar, and 20% of initial fuel consumption. The relative rate of production is indicated in blue.

**Figure 25:**
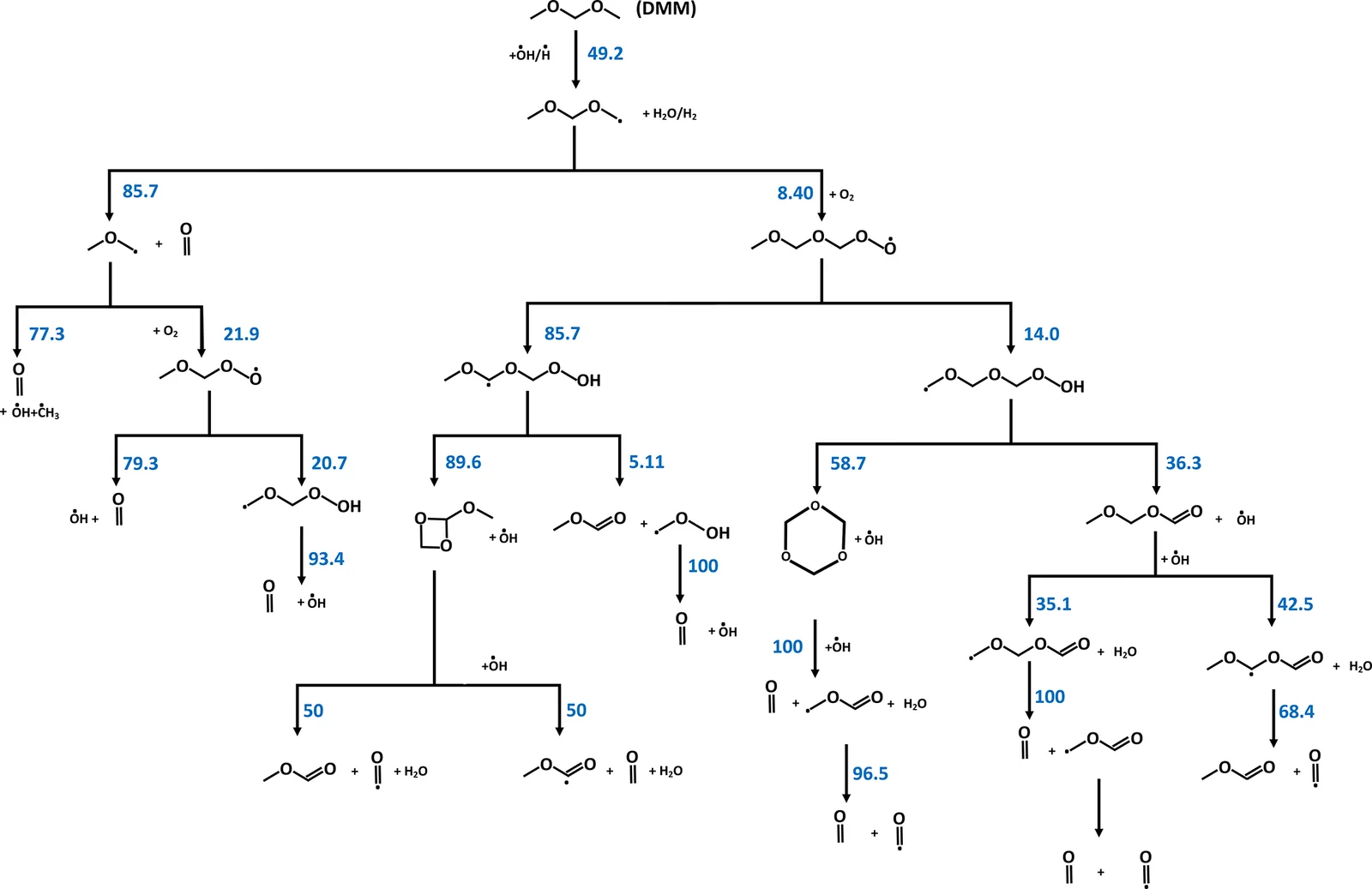
Reaction pathway for primary DMM radical formation at 373 K, *ϕ* = 1, 1 bar, and 20% of initial fuel consumption. The relative rate of production is indicated in blue.

The DMM breakdown starts with hydrogen abstraction by ȮH or Ḣ radicals and two possible pathways open depending on the type of hydrogen atom attacked, either a primary hydrogen (attached to a primary carbon atom) or a secondary hydrogen (bonded to a secondary carbon atom). A key difference between these paths is that secondary DMM radicals are consumed via unimolecular decomposition controlled by beta-scissions, where chain branching reactions are not promoted, while primary DMM radicals undergo combinations with *O*_2_ and isomerization to form hydroperoxides that, in turn, lead to chain-branching reactions that produce ȮH, contributing to the radical pool and promoting reactivity.

DMM suffers a secondary hydrogen abstraction (50.8%) that leads to the formation of a secondary DMM radical CH_3_OĊHOCH_3_ that undergoes unimolecular decomposition following the beta-scission rule, that is, the unpaired electron strengthens the adjacent bonds at the expense of the other ones further away.^72^ Thus, the *C*–*O* bond at the extreme of the molecule is broken forming methyl formate CH_3_OCHO and a methyl radical ĊH_3_.

The methyl formate suffers another hydrogen abstraction (56.5%) by ȮH or Ḣ radicals to give a methoxycarbonyl radical CH_3_OĊO that eventually decomposes to methyl radical and carbon dioxide. The hydrogen abstraction also produces a primary methyl formate radical ĊH_2_OCHO (43.5%) that decomposes to formaldehyde CH_2_O and formyl radical ĊHO. The formaldehyde reacts with Ḣ and forms a formyl radical which reacts with oxygen to form the hydroperoxyl radical HȮ_2_ and *CO*. The latter then reacts with ȮH to form *CO*_2_ and Ḣ.

Regarding the DMM primary hydrogen abstraction (Figure 25), this reaction leads to a primary DMM radical CH_3_OCH_2_OĊH_2_ (49.2%) that mainly undergoes unimolecular decomposition (85.7%) to produce methoxymethyl radical CH_3_OĊH_2_ and formaldehyde. The methoxymethyl radical can decompose to formaldehyde and ȮH (77.3%) or react with oxygen to form the peroxyl radical CH_3_OCH_2_OȮ (21.9%) which breaks down into formaldehyde and ȮH (79.3%) or suffers isomerization (20.7%), namely, an internal hydrogen shift, to form the hydroperoxide radical ĊH_2_OCH_2_OOH, which predominantly decomposes to formaldehyde and ȮH.

In appreciable amounts (8.40%), the primary DMM radical can also react with oxygen to form the peroxyl radical CH_3_OCH_2_OCH_2_OȮ which undergoes isomerization to form mainly two hydroperoxide radicals CH_3_OĊHOCH_2_OOH (85.7%) and ĊH_2_OCH_2_OCH_2_OOH (14.0%). These molecules decompose predominantly to cyclic ethers like 2-methoxy, 1,3-dioxetane, and 1,3,5-trioxane, which react with ȮH radicals to form methyl formate, formyl radical, methoxycarbonyl radical, and formaldehyde.

Other compounds that also participate in the hydroperoxide decomposition are hydroperoxy-methyl radical ĊH_2_OOH and methoxymethyl formate CH_3_OCH_2_OCHO that subsequently break down into formaldehyde, methyl formate, and formyl radicals.

It should be emphasized that in primary DMM radicals, the presence of an oxygen atom bonded to a methylene group −O–CH_2_– leads to the creation of a peroxyl radical and then a hydroperoxide (by an internal hydrogen migration). This formation of hydroperoxides in early combustion stages is important, since they decompose to ȮH radicals, which participate in oxidative processes that subsequently degrade soot precursors.^74^

### 3.4 Flame Stability Results

The Schlieren images obtained from experiments at a temperature of 373 K and pressures of 3 and 5 bar are presented in Figures 26 and 27. At 3 bar and *ϕ* = 0.8, the flame surface begins smooth and remains in this state throughout its expansion until the last frame when *R*_*f*_ = 4 cm. Two very small wrinkles can be observed above and below the left electrode at *R*_*f*_ = 3 cm that slightly grow but do not affect the general surface of the flame. At *ϕ* = 1, the flame surface remains smooth as it expands. However, wrinkles close to the electrodes start to develop at *R*_*f*_ = 2 cm and become larger at *R*_*f*_ = 3 and 4 cm, indicating that the flame is slightly more unstable. At *ϕ* = 1.2, it can be observed that at *R*_*f*_ = 2 and 3 cm, the wrinkles around the electrodes start to affect a larger portion of the flame than at *ϕ* = 1. More importantly, when the radius reaches 4 cm, the general flame surface becomes cellular, with large cracks and cell-like structures developing throughout the flame. Similarly, at *ϕ* = 1.4, when the radius reaches 3 and 4 cm, the flame is completely cellular and largely affected by instabilities, while at *R*_*f*_ = 1 cm, the flame remains smooth and at *R*_*f*_ = 2 cm, some wrinkles close to the electrodes can be observed.

**Figure 26:**
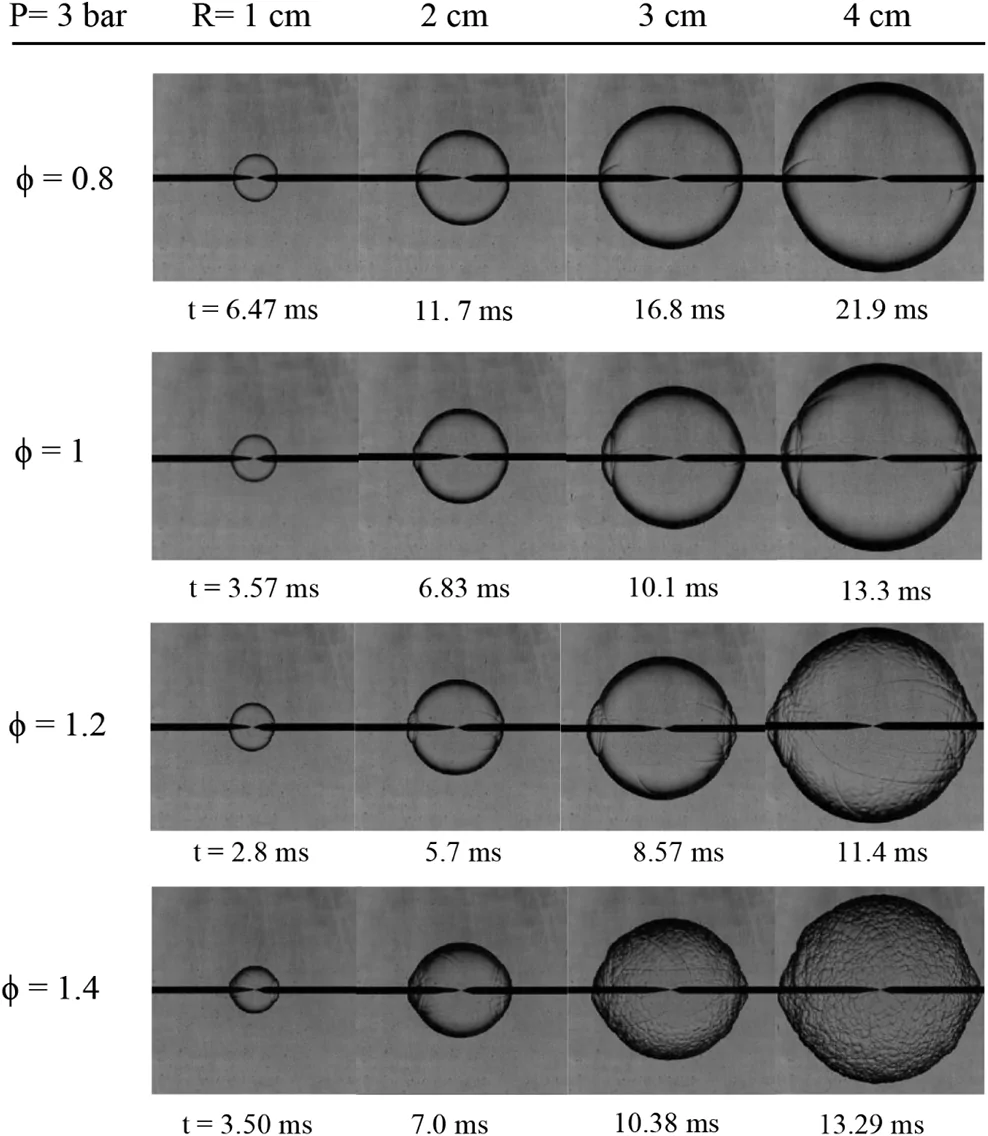
Flame morphologies of stable and unstable flames at 373 K, 3 bar, and equivalence ratios from 0.8 to 1.4.

**Figure 27:**
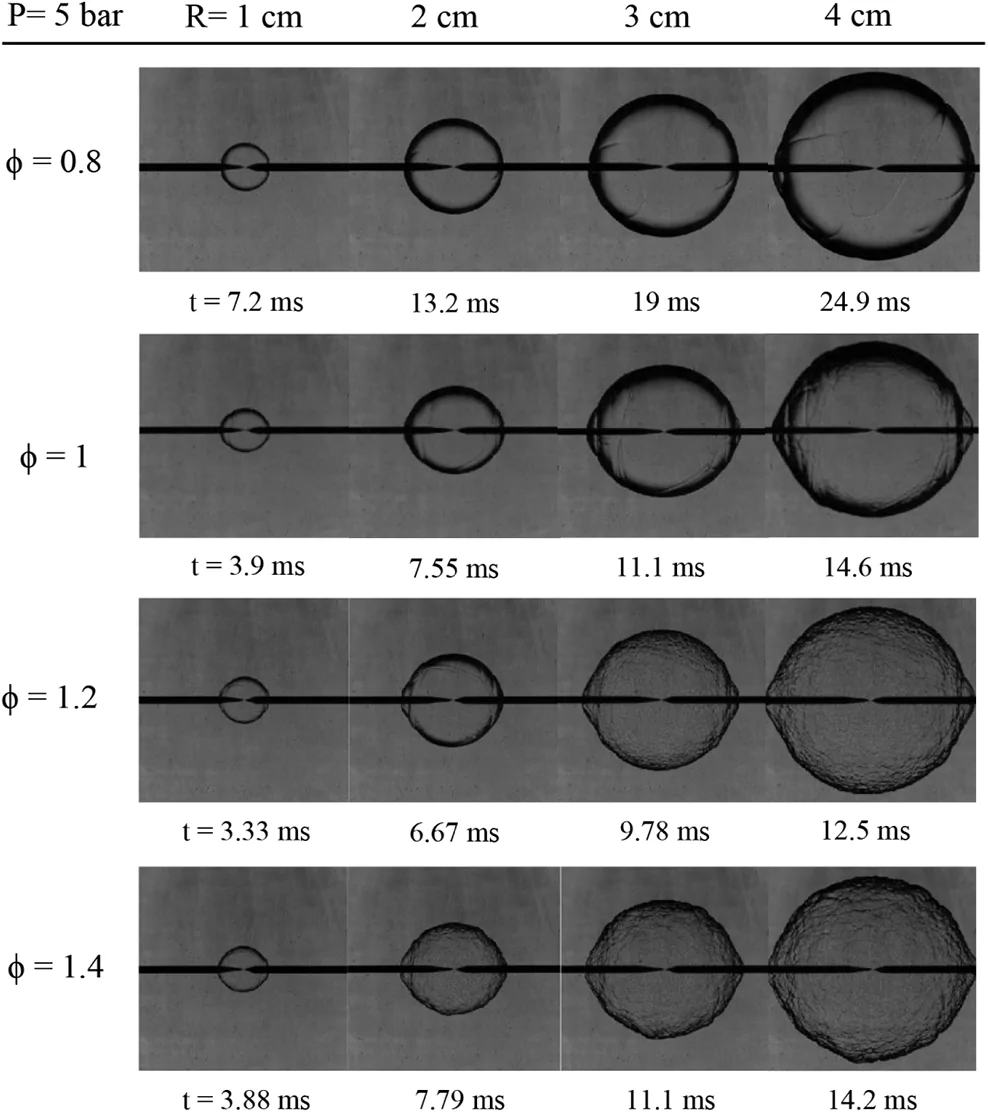
Flame morphologies of stable and unstable flames at 373 K, 5 bar, and equivalence ratios from 0.8 to 1.4.

In Figure 27, the flame morphology can be observed at 373 K, 5 bar, and several equivalence ratios. At *ϕ* = 0.8, the flame expands and remains smooth. Some minor wrinkles can be observed at *R*_*f*_ = 2 cm that grow and become larger at *R*_*f*_ = 4 cm. At *ϕ* = 1, it can be noted that large cracks occur at *R*_*f*_ = 3 cm, and the cellularity is intensified at *R*_*f*_ = 4 cm. At *ϕ* = 1.2, the flame surface at *R*_*f*_ = 3 and 4 cm becomes fully affected by cellularity and some large cracks on the flame surface can be observed earlier at *R*_*f*_ = 2 cm. Finally, at *ϕ* = 1.4, the instability is intensified and the flame becomes fully wrinkled at *R*_*f*_ = 2 cm and beyond.

The development of cellular structures, as mentioned in Section 3.1, affected the laminar flame speed calculation. Particularly at 3 bar and equivalence ratios of 1.2, 1.3, 1.4, and 1.5, the onset of intense cellular instabilities started at 3.5, 2.3, 2.1, and 1.9 cm, respectively. At 373 K and 5 bar, the flame speed calculation was affected by cellular structures in an earlier propagation state. At equivalence ratios of 1, 1.1, 1.2, 1.3, and 1.4 and 5 bar, the cellularity onset occurred at 2.9, 2.4, 2, 1.5, and 1.1 cm, respectively. Clearly, flames at higher pressures and equivalence ratios become significantly more unstable, which triggers the earlier formation of cellular structures.

As mentioned in the introduction, the three main mechanisms that affect flame stability are diffusional-thermal (characterized by the effective Lewis number), hydrodynamic (quantified by density ratio and thermal-gradient flame thickness), and body forces. The total influence of the combined mechanism defines the critical radius at which a flame becomes unstable.^54^ Hence, to separately quantify the effect of each mechanism, the effective Lewis number, adiabatic flame temperature, flame thickness, and density ratio are estimated with numerical simulations.

The calculated effective Lewis number is presented in Figure 28 at 1 bar, temperatures of 373, 423, and 473 K, and equivalence ratios from 0.7 to 1.7. The calculated effective Lewis number did not change with increasing pressure, since both the thermal diffusivity α and mass diffusivity *D*_*ij*_ are inversely proportional to pressure and the ratio remains constant.^52^ A small decrease in the effective Lewis number was calculated with increasing temperature, indicating that the flame becomes slightly more thermodiffusively unstable at higher temperatures. More prominent is the reduction of *Le*_*eff*_ with increasing equivalence ratios.

**Figure 28:**
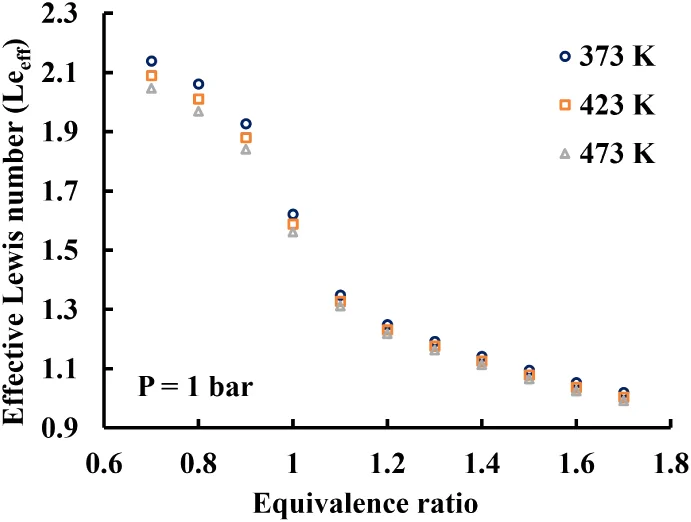
Effective Lewis number versus equivalence ratio for DMM flames at 1 bar and 373, 423, and 473 K.

Generally, in premixed flames, preferential diffusion is controlled by the deficient reactant. If the deficient reactant is highly diffusive, the composition of the reaction zone becomes richer and both the temperature and burning rate increase. Conversely, if the deficient reactant is weakly diffusive, its concentration in the reaction zone decreases, which reduces the burning rate.^52^ For this reason, hydrogen flames are extremely unstable on the lean side and become stable on the rich side, whereas alkane flames such as propane exhibit the opposite behavior.^53,58,75^

DMM is a heavier molecule than oxygen, and thus, its mass diffusivity is lower than that of oxygen (0.125 compared to 0.289 cm^2^/s at 1 bar, 373 K, and *ϕ* = 0.7). As a result, in lean DMM/air flames, DMM is the deficient reactant, and it increases the effective Lewis number, making the flame more thermodiffusively stable. On the contrary, in rich DMM/air flames, oxygen becomes the limiting reactant, and due to its relatively higher mass diffusivity, it decreases the effective Lewis number, making the flame more thermodiffusively unstable.

As can be observed in Figure 28, at *ϕ* = 0.7, 1 bar, and 373 K, *Le*_*eff*_ is 2.14, indicating that heat diffusion is significantly higher than the mass diffusion, and this mechanism has a stabilizing effect on the flame (*Le*_*eff*_ > 1). When the equivalence ratio increases, the effective Lewis number decreases significantly, particularly above *ϕ* = 1. Therefore, there is an important reduction in the capacity of the thermodiffusive mechanism to dampen perturbations at high equivalence ratios, and the flame becomes more susceptible to diffusional-thermal instabilities.

At 1 bar, 373 K, and *ϕ* = 1.3, 1.5, and 1.7, the calculated effective Lewis numbers were 1.19, 1.09, and 1.02, respectively. At *ϕ* = 1.7, 1 bar, and temperatures of 423, and 473 K, the calculated effective Lewis numbers were 1.00, and 0.99, respectively. Therefore, the numerical results indicate that, at most of the rich conditions, the limit for the thermodiffusive instability of *Le*_*eff*_ = 1 is not reached. Instead, *Le*_*eff*_ is significantly closer to the instability threshold, and thus, the stabilization effect of the thermodiffusive mechanism is progressively reduced.

Even though the numerical *Le*_*eff*_ does not go below 1 under most of the rich conditions, it is important to mention that the experimentally measured Markstein length does become negative at higher equivalence ratios. At 373 K and 1 bar, the Markstein length values obtained at *ϕ* = 1.6 and 1.7 were −0.364 ± 0.0211 and −0.733 ± 0.0152 mm, respectively. At 423 K and 1 bar, the Markstein length values were −0.222 ± 0.0116 and −0.665 ± 0.0186 mm at *ϕ* = 1.6 and 1.7, respectively. Moreover, at 473 K, 1 bar, and *ϕ* = 1.6 and 1.7, the Markstein length values were −0.109 ± 0.0173 and −0.325 ± 0.02 mm, respectively. This indicates that the thermodiffusive contribution becomes destabilizing, and thus, the actual effective Lewis number of the mixture is sufficiently below 1 to lower the Markstein length to negative values. Consequently, the calculated values of *Le*_*eff*_ under rich conditions are slightly overestimated. This small discrepancy can arise from the fact that *Le*_*eff*_ is an approximation that considers a weighted average of the diffusion rates of oxygen and fuel at the initial conditions, while real flames involve multiple reactive components with variable physicochemical properties and diffusion rates. Therefore, the conditions at which the Markstein length becomes negative are considered thermodiffusively unstable.

Regarding hydrodynamic instability, this mechanism is enhanced by an increase in the density ratio (*σ*) and a decrease in thermal-gradient flame thickness (δ_*tg*_).^54^ The effect of equivalence ratio, temperature, and pressure on adiabatic flame temperature, density ratio, and thermal-gradient flame thickness is shown in Figure 29. An increase in initial temperature *T*_*u*_ increases the adiabatic flame temperature and decreases both the unburned gas density *ρ*_*u*_ and the burned gas density *ρ*_*b*_. However, the unburned gas density decreases more than the burned gas density, thereby reducing the density ratio.

**Figure 29:**
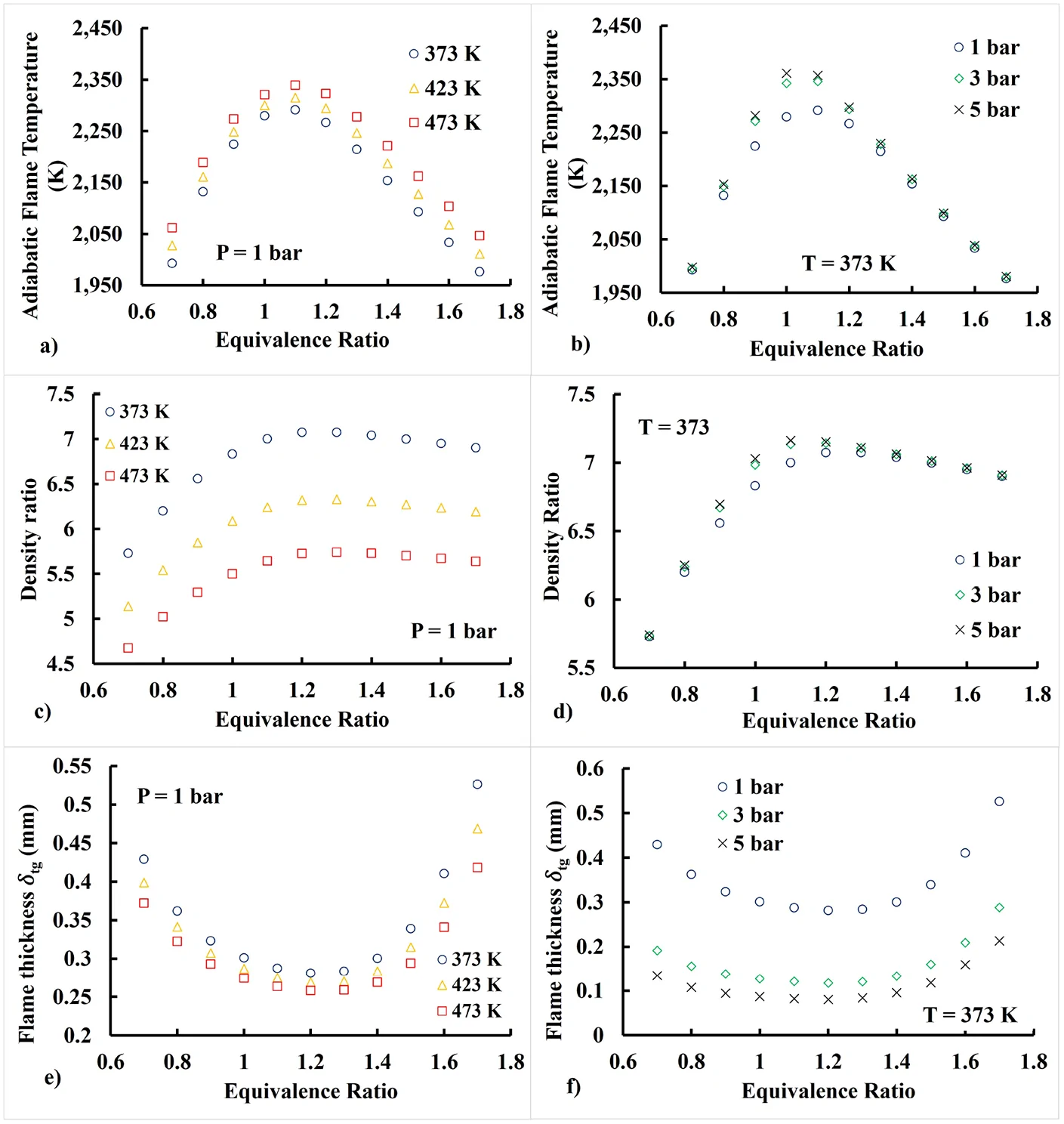
Effect of equivalence ratio, temperature, and pressure on adiabatic flame temperature, density ratio, and thermal-gradient flame thickness.

Similarly, an increase in pressure rises the adiabatic flame temperature, and both the burned gas and unburned gas densities go up, increasing the density ratio, particularly in the range of equivalence ratios 0.9–1.2, as shown in Figure 29d.

The thermal-gradient flame thickness is sensitive to changes in temperature and pressure, as shown in Figure 29e and f. Both an increase in temperature and an increase in pressure reduce the thermal-gradient flame thickness by accelerating the reaction rate.^52^ However, the effect of the pressure on the flame thickness is substantially more pronounced.

At an equivalence ratio of 0.7, the thermal-gradient flame thickness is relatively high and decreases with increasing equivalence ratio until *ϕ* = 1.2 is reached. At this point, the density ratio is the highest. Thus, the combined effect of low thermal-gradient flame thickness and a high density jump contributes to the amplification of hydrodynamic perturbations. Above *ϕ* = 1.3, the flame thickness starts to increase. However, the density jump remains high, reducing the damping effect of a thicker flame on disturbances.

Experimentally, an earlier formation of cellular structures was observed at increasing pressures. For instance, at 373 K and *ϕ* = 1.2, at 1 bar, no cellularity was observed, while at 3 and 5 bar, intense cellularity was observed at *R*_*f*_ = 3.5 and 2 cm, respectively. Since the effective Lewis number remains pressure-independent, this change is not attributed to a decrease in thermodiffusive stability. In contrast, an increase in pressure notoriously decreases the thermal-gradient flame thickness (from 0.28 mm at 1 bar to 0.117 and 0.08 mm at 3 and 5 bar, respectively) while a significant density ratio is maintained (7.07, 7.14, and 7.15 at 1, 3, and 5 bar, respectively). Therefore, this trend is consistent with a more pronounced hydrodynamic instability. Moreover, the stabilizing effect of stretch is weakened, since the Markstein length decreases from 0.69 ± 0.0216 mm at *ϕ* = 1.2 and 1 bar to 0.248 ± 0.02 and 0.156 ± 0.0157 mm at 3 and 5 bar, respectively.

In addition, at a fixed temperature and pressure, the observed increasing instability at higher equivalence ratios is consistent with the combined contribution of weakened stabilization from the stretch and thermodiffusive effects, together with higher density ratios that enhance the growth of hydrodynamic instabilities. For instance, at 3 bar and 373 K, at *ϕ* = 0.8, no cellularity was observed while at *ϕ* = 1.4, the flame was fully cellular at *R*_*f*_ = 3 and 4 cm. At *ϕ* = 0.8, the calculated Lewis number, Markstein length, and density ratio were 2.05, 0.505 mm, and 6.24, respectively. At *ϕ* = 1.4, the same parameters reached values of 1.13, 0.107 mm, and 7.06, respectively.

It can be concluded that both the thermodiffusive and hydrodynamic mechanisms play an important role in flame instability, particularly at higher pressures and equivalence ratios.

## 4 Conclusions

DMM/air mixtures were ignited at several temperatures and pressures in a constant-volume combustion vessel. The flame front propagation was captured and recorded using a Schlieren imaging technique to calculate the unstretched laminar flame speed. The results indicated that the maximum flame speeds at 1 bar and 373, 423, and 473 K were 63.4, 77.5, and 91.8 cm/s, respectively. In addition, at 373 K and 3 and 5 bar, the measured maximum values were 49.8 and 43.2 cm/s at *ϕ* = 1.2 and *ϕ* = 1.1, respectively. At 423 K and 3 bar, the highest measured laminar flame speed value was 61.5 cm/s at *ϕ* = 1.2. At 423 K and 5 bar, the flame speed was measured up to *ϕ* = 1, for which a value of 50.4 cm/s was determined. An increase in DMM laminar flame speed with increasing temperature and decreasing pressure was observed.

The experimental DMM laminar flame speed values were compared to results from 1D laminar flame speed simulations using various kinetic mechanisms. The mechanisms from Jacobs et al.^14^ and Shrestha et al.^13^ generally showed the best performance. Closer agreement was found between experimental and simulated results at 3 and 5 bar as well as 1 bar and 373 K. However, the difference increased at higher temperatures.

Good agreement was found when comparing the experimental results at 373 K and 1 bar with those reported by Shrestha et al.^13^

A sensitivity analysis was carried out at three equivalence ratios (0.7, 1, and 1.7) and demonstrated that the laminar flame speed is significantly sensitive to chain-branching and chain-terminating reactions that produce or consume reactive radicals such as ȮH, Ö, and Ḣ, since they play a vital role in successive kinetic reactions that decompose the fuel into final combustion products. Under all conditions, the most influential reaction has been found to be reaction R5 O_2_ + Ḣ = Ö + ȮH, which indicates the fundamental role of H/O chemistry on DMM flames. Under lean conditions (*ϕ* = 0.7), reactions involving O_2_, HȮ_2_, and fuel fragments such as CO, methyl radical ĊH_3_, and formyl radical HĊO were among the most sensitive reactions. At rich conditions (*ϕ* = 1.7), the fuel chemistry became substantially more important than in the lean case, and several more reactions involving DMM and fuel fragments were identified, while reactions involving O_2_ and HȮ_2_ became less influential. The sensitivity coefficients were not substantially affected by temperature. Nonetheless, pressure significantly affected three-body chain-terminating reactions.

The reaction pathway analysis indicated that DMM breakdown started with the formation of primary and secondary DMM radicals by the attack of ȮH and Ḣ radicals that subtract a hydrogen atom from the DMM molecule. Secondary DMM radicals are consumed via unimolecular decomposition reactions that follow the beta-scission rule and hinder the formation of reactive radicals, while the primary DMM radicals undergo combinations with oxygen and isomerization to form hydroperoxides that lead to chain-branching reactions that produce ȮH radicals.

The flame stability was found to be substantially affected by an increase in pressure and equivalence ratio, due to increased diffusional-thermal and hydrodynamic instability, while an increase in temperature only slightly reduced the calculated effective Lewis number and thus the thermodiffusive stability. The development of cellular structures did not occur at 1 bar. Nevertheless, intense flame instability was observed at 3 and 5 bar, and it affected the flame speed calculation at these conditions. The numerically calculated effective Lewis number reached values very close to unity for most conditions at high equivalence ratios. The lowest calculated *Le*_*eff*_ was 0.99 at 1 bar, 473 K, and *ϕ* = 1.7. However, the experimentally measured Markstein length reached negative values at high equivalence ratios, indicating a destabilizing response of the flame to stretch that originated from a thermodiffusive contribution. Thus, it was concluded that the increase in equivalence ratio led to thermodiffusive instability. Besides, the density ratio was found to increase with increasing equivalence ratios (until *ϕ* = 1.1–1.2), a favorable condition for hydrodynamic instability. Moreover, it was observed that the increase in pressure significantly reduced the thermal-gradient flame thickness and thus intensified flame hydrodynamic instability. In addition, it was observed that the Markstein length was positive at low equivalence ratios, indicating a stabilizing effect of stretch on the flame, while at higher equivalence ratios and pressures, the Markstein length decreased significantly, reaching negative values and thereby generating a destabilizing stretch effect that contributed to intensified instability and cellularity.

## Supplementary Material



## Notes

Table S1. Experimental flame speed: includes the 3-test average laminar flame speed values measured at each condition, and presented in the paper plots, with the corresponding uncertainties in cm/s, and the correlation used (Markstein or Kelley and Law). Table S2. Flame speed correlations: includes the results from both flame speed correlations (Markstein and Kelley and Law). Table S3. Simulated flame speed: includes the simulated laminar flame speed values from different kinetic mechanisms, in cm/s. Table S4. Markstein length: includes the measured Markstein lengths presented in the paper plots, with the corresponding uncertainties in mm. Table S5. Markstein length correlations: includes the Markstein length values from both correlations (Markstein and Kelley and Law), in mm. Table S6 Stability results: includes the calculated effective Lewis number, Zel’dovich number, preheat flame thickness (mm), thermal-gradient flame thickness (mm), adiabatic flame temperature (K), and density ratio, calculated from the simulations using the Jacobs et al.^14^ mechanism (XLSX)

## Abbreviations

1D: one-dimensional
α: thermal diffusivity
δ_*tg*_: thermal-gradient flame thickness
δ_*l*_: preheat flame thickness
$\delta_{S_{L}}$: total laminar flame speed uncertainty
ϕ: equivalence ratio
κ: stretch rate
ρ_*u*_: unburned gas density
ρ_*b*_: burned gas density
*σ*: density ratio
*A*: flame surface area
*B*_*T*_: systematic uncertainty due to temperature variations
*B*_ϕ_: systematic uncertainty due to equivalence ratio variations
C: carbon atom
CFD: computational fluid dynamics
CO_2_: carbon dioxide
CVCC: constant-volume combustion chamber
D_*ij*_: binary diffusivity coefficient
DMC: dimethyl carbonate
DME: dimethyl ether
DMM: dimethoxymethane (C_3_H_8_O_2_)
E_*a*_: activation energy
EGR: exhaust gas recirculation
Gt: gigatons
HVO: hydrotreated vegetable oil
*L*_*b*_: Markstein length
*Le*: Lewis number
*Le*_*eff*_: effective Lewis number
N: number of experiments
N_2_: nitrogen
NH_3_: ammonia
ODE: ordinary differential equation
OME: oxymethylene ether
OME_3_: oxymethylene ether 3
P: pressure
*P*_*air*_: partial pressure of air
*P*_*initial*_: initial pressure of the experiment
*P*_*fuel*_: partial pressure of fuel
PODE: polyoxymethylene dimethyl ether
PRF: primary reference fuel
*R*_*f*_: flame radius
RPA: reaction path analyzer
*S*_*b*_: stretched burned gas laminar flame speed
$S_{b}^{◦}$: unstretched burned gas laminar flame speed
$S_{u}^{◦}$: unstretched unburned gas laminar flame speed
$S_{u_{sim}}^{◦}$: simulated laminar flame speed
t: time
*T*_*u*_: unburned gas temperature
*T*_*ad*_: adiabatic flame temperature (burned gas temperature)
*t*_0.95_: Student’s *t* for a 95% confidence level
$S_{S_{L}}$: standard deviation in laminar flame speed
(*A*/*F*)_ϕ=1_: stoichiometric air-to-fuel ratio (by mass)
MW: molecular weight
*Ze*: Zel’dovich number
